# Small Molecule‐Induced Alterations of Protein Polyubiquitination Revealed by Mass‐Spectrometric Ubiquitome Analysis

**DOI:** 10.1002/anie.202508916

**Published:** 2025-06-26

**Authors:** Siska Führer, Kai Gallant, Farnusch Kaschani, Markus Kaiser, Petra Janning, Herbert Waldmann, Malte Gersch

**Affiliations:** ^1^ Chemical Genomics Centre Max Planck Institute of Molecular Physiology Otto‐Hahn‐Str. 15 44227 Dortmund Germany; ^2^ Department of Chemical Biology Max Planck Institute of Molecular Physiology Otto‐Hahn‐Str. 11 44227 Dortmund Germany; ^3^ Department of Chemistry and Chemical Biology TU Dortmund University Otto‐Hahn‐Str. 4a 44227 Dortmund Germany; ^4^ Faculty of Biology Analytics Core Facility Essen (ACE) ZMB University of Duisburg‐Essen Essen Germany

**Keywords:** Biological activity, Drug discovery, Protein modifications, Proteomics, Ubiquitin proteasome system

## Abstract

Small molecules that alter protein ubiquitination are emerging as therapeutics due to their ability to modulate targets previously deemed undruggable. These compounds comprise PROTACs, molecular glue degraders, and DUB inhibitors, among others. However, methods for the proteome‐wide monitoring of compound‐induced changes in protein polyubiquitination, which may also detect non‐degradative modifications, are lacking. Here, we report the utilization of polyubiquitin enrichment coupled to mass spectrometry to monitor small molecule‐induced changes in cellular protein ubiquitination. We established enrichment through tandem ubiquitin binding entities (TUBEs) following semi‐denaturing cell lysis and devised an elution protocol compatible with downstream LC‐MS/MS analysis. We demonstrate the broad applicability of the workflow by assessing ubiquitination changes induced by a PROTAC, a p97 inhibitor, and deubiquitinase inhibitors. Application of the assay to compounds inhibiting the deubiquitinase USP7 revealed the induction of non‐degradative ubiquitination on the E3 ligase UBE3A. Collectively, we established a versatile proteomics method to facilitate the direct investigation of cellular polyubiquitination, with high relevance for the identification and characterization of protein degraders, stabilizers, and other molecules with ubiquitin‐mediated bioactivity.

## Graphical abstract

To monitor small molecule‐induced changes in protein polyubiquitination in an unbiased manner, we employ enrichment through tandem ubiquitin binding entities coupled to LC‐MS/MS analysis (TUBE‐MS). By applying the workflow to PROTAC, p97‐ and deubiquitinase‐inhibitor treatment of cells, we demonstrate its broad applicability and reveal the induction of non‐degradative ubiquitination on the E3 ligase UBE3A upon inhibition of the deubiquitinase USP7.

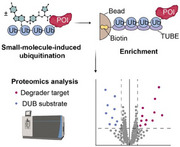

## Introduction

**Figure 1:**
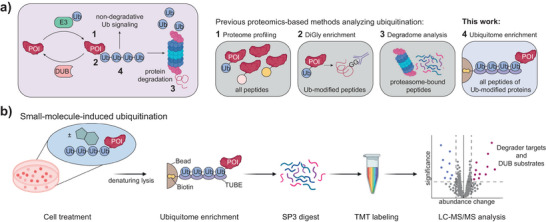
Experimental design of TUBE PDs followed by mass spectrometry for ubiquitome analysis. a) Schematic of the Ub system, highlighting degradative and non‐degradative polyubiquitin modifications (*left*). Four complementary proteomics‐based methods for ubiquitome analysis (*right*), covering proteome profiling (1), diGly enrichment (2), degradome analysis (3), or polyubiquitin PD via TUBEs (4). Measured molecular properties are stated on the right and are indicated in the system with numbers in bold. b) Schematic of the workflow described in this manuscript for the proteome‐wide assessment of compound‐induced changes in protein ubiquitination. TUBE‐mediated enrichment of ubiquitinated species is followed by SP3 digest, TMT labeling, and LC‐MS/MS analysis for quantification of polyubiquitinated proteins.

Ubiquitin (Ub) is a key post‐translational modifier that regulates diverse cellular processes. The complexity of the Ub system arises from the versatile topologies of Ub‐mediated modifications, ranging from monoubiquitination to differently linked Ub chains. Although polyubiquitination often targets proteins for degradation via the proteasome, it also plays crucial non‐degradative roles (Figure 1a), e.g., in protein trafficking, immune signaling, and the DNA damage response.^[^ ^1^, ^2^, ^3^, ^4^ ^]^ The contributions of Ub to malignant processes such as cancer, neurodegeneration, and inflammation make enzymes mediating ubiquitination (E3 ligases) as well as deubiquitinating enzymes (DUBs) attractive targets for therapeutic innovations.^[^ ^5^, ^6^, ^7^ ^]^

One of these approaches comprises small‐molecule‐induced protein degradation^[^ ^8^, ^9^ ^]^ in which proteolysis targeting chimeras (PROTACs) or molecular glue degraders induce proximity between an E3 ligase and a neosubstrate.^[^ ^8^, ^10^, ^11^, ^12^, ^13^ ^]^ A complementary approach of interfering with Ub signaling is the inhibition of deubiquitinases, causing decreased cleavage of Ub modifications on DUB substrate proteins. This strategy allows for amplification of Ub‐mediated signaling without a ligand directly binding the substrate. Multiple molecules utilizing targeted protein degradation and DUB inhibition are being investigated in clinical trials, underscoring their potential for therapeutic innovation and for addressing targets previously deemed undruggable.^[^ ^14^, ^15^ ^]^ These developments stress the demand for robust methods to facilitate a proteome‐wide monitoring of compound‐induced changes in protein polyubiquitination.

A challenge associated with the discovery and characterization of degraders and Ub‐interfering molecules is the efficient detection of Ub chain formation on proteins.^[^ ^16^ ^]^ Whereas classical inhibitor‐based drug discovery can often rely on direct detection of enzymatic activities, the unbiased investigation of small molecule‐induced ubiquitination mainly relies on indirect detection methods through mass spectrometry.^[^ ^16^ ^]^ Three main proteomics‐based approaches have emerged: 1) Whole proteome analysis is routinely employed to follow up on phenotypic screens (Figure 1a, method 1), with changes in protein levels upon compound treatment serving as a proxy for proteasome recruitment after ubiquitination.^[^ ^11^, ^12^, ^13^, ^16^, ^17^ ^]^ However, whole proteome analysis is neither capable to differentiate compound‐related primary from downstream effects, nor able to recognize non‐degradative ubiquitination;^[^ ^16^, ^18^ ^]^ 2) Changes in protein ubiquitination can be assessed by enriching individual peptides comprising the characteristic diGly remnant formed after tryptic digest^[^ ^16^, ^19^ ^]^ (Figure 1a, method 2). This procedure uniquely measures site‐specific ubiquitination of target proteins (typically not possible with whole proteome analysis^[^ ^16^ ^]^) but lacks insights into the presence of Ub chains. 3) In addition, degradome analysis, for which peptides of digested proteins are captured inside proteasomes, can efficiently be used to probe the cellular degradation landscape^[^ ^20^ ^]^ (Figure 1a, method 3).

However, some of these workflows do not detect non‐degradative polyubiquitin and, importantly, all lack the ability to directly detect the presence of Ub chains. We therefore sought to develop a robust mass spectrometry‐based method to quantitatively monitor small‐molecule‐induced changes in cellular protein polyubiquitination. Taking the very low global ubiquitination site occupancies into account,^[^ ^16^, ^21^ ^]^ we utilized tandem ubiquitin binding entities (TUBEs) to selectively enrich polyubiquitinated proteins irrespective of their Ub chain linkages.^[^ ^22^, ^23^ ^]^ These tools are broadly used for immunoblot‐based readouts to characterize polyubiquitin dynamics. Few studies have showcased the potential of their coupling to mass spectrometry, yet were hampered by comparably low protein identification rates and sensitivity.^[^ ^24^, ^25^, ^26^, ^27^, ^28^ ^]^

Building on these and related workflows,^[^ ^29^ ^]^ we here report a broadly applicable method for polyubiquitin enrichment coupled to mass spectrometry (Figure 1b). Our TUBE‐MS workflow covered investigations of reagents, lysis conditions, and multiplexing to reduce the background of Ub‐binding proteins, to increase sensitivity through preservation of Ub modifications, and to elevate detection rates in the mass‐spectrometric readout. We thereby unite the broad applicability of a whole proteome analysis and the specificity of a diGly‐enrichment, establishing a method suitable for investigating small‐molecule‐induced degradative and non‐degradative polyubiquitination. Application of the assay to a USP7 inhibitor led to the discovery of non‐degradative ubiquitination on the UBE3A E3 ligase.

## Results

### Cellular Polyubiquitin Enrichment

**Figure 2:**
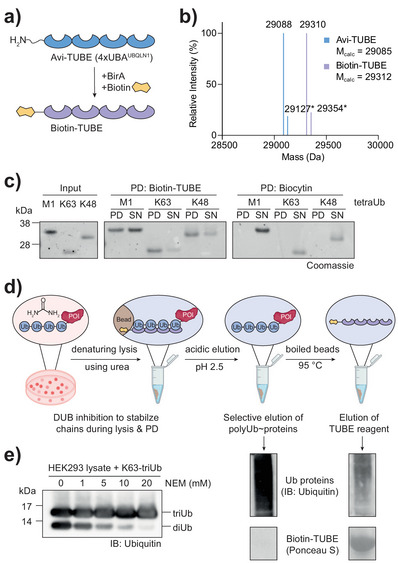
TUBE PDs with compatibility to LC‐MS/MS analysis. a). Schematic of BirA‐catalyzed biotinylation of the N‐terminally Avi‐tagged 4xUBA^UBQLN1^ TUBE protein, which was used as bait for the enrichment of polyubiquitinated species in this work. b) Overlay of two intact protein mass spectra recorded of the purified TUBE reagent before (Avi‐TUBE, blue) and after (Biotin‐TUBE, purple) biotinylation. Found and calculated masses are given in Dalton. ’*’ indicates gas‐phase acetonitrile adducts of the proteins. c) In vitro PD of differently linked tetraubiquitin (tetraUb) chains using Biotin‐TUBE compared to Biocytin as control. PD fractions are depicted next to the respective supernatant (SN) fractions. d) Schematic of the optimized PD from lysate. Cell lysis was performed under semi‐denaturing conditions to deplete Ub‐binding proteins while maintaining PD efficiency. Acidic elution of enriched Ub‐modified proteins with 100 mM glycine pH 2.5 allowed separate liberation of cargo and bait protein off streptavidin beads, as confirmed by eluate analysis shown below (full gels are shown in Figure S1a). e) Titration of *N‐*ethylmaleimide (NEM) for complete inactivation of DUBs. K63‐triUb chains were spiked into HEK293 lysate lysed in buffer with indicated amounts of NEM, and samples were analyzed by Western blot. Uncropped versions of all gels and blots are shown in the Supporting Information.

We intended our workflow to be based on the direct recognition of polyubiquitin chains, thus complementing previously established approaches (Figure 1a, method 4). To efficiently enrich the entire polyubiquitome, we chose as bait the well‐characterized TUBE protein comprised of four repeats of the Ub‐binding UBA domain derived from ubiquilin‐1.^[^ ^22^ ^]^ This reagent can potently enrich chains of all Ub chain linkages.^[^ ^26^ ^]^ We site‐specifically biotinylated the recombinantly expressed TUBE reagent using BirA^[^ ^30^ ^]^ on an N‐terminal Avi‐tag (Figure 2a,b) to facilitate immobilization onto beads. We validated the TUBE's ability to enrich differently linked Ub chains in vitro over a biocytin control, showing successful pulldown (PD) of free Ub chains (Figure 2c). This experiment was carried out with K48 und K63 chains, which together account for >90% of the cellular polyUb pool.^[^ ^31^, ^32^ ^]^

We next defined a protocol for polyubiquitin enrichment from cells (Figures 2d and S1) based on semi‐denaturing cell lysis and washing conditions with 4 M urea. These were recently introduced for the profiling of protein ubiquitination with a bacterial monoubiquitin binder,^[^ ^29^ ^]^ and serve to separate ubiquitinated from unmodified proteins as well as from Ub‐binding proteins. In addition, we aimed to preserve Ub chains during cell lysis by completely inhibiting cellular deubiquitinases. Exploring different combinations of DUB‐inhibiting buffer additives (Figure S2a), we focused on *N‐*ethylmaleimide (NEM) to inhibit cysteine‐based DUBs. Through spiking recombinant triubiquitin chains into HEK293 lysate, we established an NEM concentration of 20 mM to be essential for full DUB inhibition upon cell lysis (Figure 2e).

Magnetic streptavidin beads were used to minimize variations during sample handling and to allow automatization. An advantage of biotin as a tag was the quantitative, straightforward, and modular preparation of the TUBE‐bead‐assembly immediately before use, without the necessity of non‐site‐selective covalent ligations or concerns about long‐term stability of immobilized reagents. We confirmed that the PD under semi‐denaturing conditions^[^ ^29^ ^]^ preserved PD efficiency (Figures S1a,b, and S3a).

In view of a sensitive mass spectrometric readout, we aimed to reduce the amount of TUBE protein in the final eluate. We identified that ubiquitinated proteins are selectively liberated from the beads during rounds of acidic elution, while the interaction of biotin and streptavidin persisted (Figure 2d, see presence of cellular polyubiquitin and concomitant absence of TUBE reagent in elution fraction). This finding demonstrates that the workflow enabled the enrichment and separation of ubiquitinated proteins.

### Unbiased Mass‐Spectrometric Readout of Compound‐Induced Protein Ubiquitination

**Figure 3:**
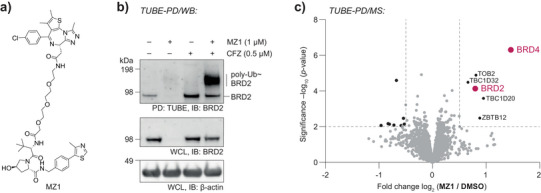
Optimized protocol for polyubiquitin PD coupled to mass spectrometry detects MZ1 PROTAC‐mediated increases in BRD protein ubiquitination. a) Structure of the PROTAC MZ1, consisting of a handle engaging the VHL E3 ligase and the BRD‐binder JQ1. b) Immunoblot‐based evaluation of polyubiquitin PD eluates. Jurkat cells were treated with degrader MZ1 for 4 h with proteasomal inhibition using CFZ for 5 h, including 1 h of preincubation, where indicated. Cell lysates were then subjected to polyubiquitin enrichment and Western blotting (WB/IB), showing enriched ubiquitination of BRD2. WCL, whole cell lysate. Uncropped versions of all blots are shown in the Supporting Information. c) Volcano plot of protein abundances after proteomic analysis of polyubiquitin PDs from Jurkat cells treated with MZ1 or DMSO. The significant enrichment of the MZ1 targets BRD2 and BRD4 is highlighted. PDs were performed using lysates from cells with proteasome (0.5 µM CFZ) and p97 (5 µM NMS‐873) inhibition. Proteomics data were recorded in quadruplicate from independently treated cell samples and were analyzed using FragPipe as described in the methods section.

Next, we applied the PD procedure to a known degrader to serve as a proof of principle. Here, we chose the well‐characterized PROTAC MZ1 (Figure 3a), which causes selective ubiquitination and degradation of bromodomain‐containing BET family members.^[^ ^33^ ^]^ We treated Jurkat cells with MZ1 after preincubation for 1 h with the proteasomal inhibitor Carfilzomib (CFZ) to preserve ubiquitinated BRD proteins from being degraded (Figure S2c). We then refined the PD guided by the detection of ubiquitination of the degrader target BRD2 by immunoblotting, which occurred in parallel to the above‐described workflow development. Probing PD eluates from Jurkat cell lysates, our conditions enabled the robust detection of polyubiquitinated BRD2 in an MZ1‐ and CFZ‐dependent manner, as evident from a high molecular weight signal in immunoblots alongside a minor signal caused by unmodified BRD2 (Figures 3b and S2d). The co‐enrichment of proteins not bearing Ub is commonly observed,^[^ ^22^, ^34^ ^]^ a direct result of the very low site occupancy of ubiquitination,^[^ ^21^ ^]^ and a consequence of the balance between enrichment efficiency and selectivity. Lower urea concentrations yield more nonspecific TUBE‐enrichments, whereas higher urea concentrations result in less ubiquitinated proteins being bound. Our findings demonstrate that a concentration of 4 M urea provides an optimal balance between these two factors (Figure S3a), enabling efficient and specific enrichment of cellular polyubiquitin. The signal of ubiquitinated BRD2 could be further enhanced when combining CFZ with NMS‐873, which inhibits the ATP‐dependent unfoldase p97 (also termed VCP), for pretreatment of cells (Figure S2b). This PD procedure allowed progression from a target‐specific immunoblot‐based readout to an unbiased mass spectrometry‐based analysis of PD fractions.

To prepare eluates for mass spectrometric analysis, we used the single pot, solid phase, sample preparation (SP3) protocol, covering sample cleanup and protein digestion.^[^ ^35^ ^]^ Resulting peptides were subjected to tandem mass tag (TMT) isotopic labeling for multiplexing, which in turn also increased proteome depth and facilitated accurate quantification of less abundant proteins. In contrast, previous attempts to obtain data through label‐free quantification experiments were hampered by missing values, which is in line with the expected sample complexity of a cellular polyubiquitin PD.

The TUBE‐MS workflow allowed quantification of 6084 proteins and demonstrated MZ1 target proteins BRD2 and BRD4 being significantly enriched (Figure 3c and Table S1), in line with their expected ubiquitination in PROTAC‐treated cells. In addition, increased ubiquitination was observed for the anti‐proliferative protein TOB2, the Rab‐GAP TBC domain‐containing proteins TBC1D20/32, and the Zinc finger and BTB domain‐containing protein 12 (ZBTB12), which have not been previously reported in the context of MZ1. Notably, eluates contained Ub as the most abundant protein, confirming the enrichment and successful separation of the TUBE reagent (Figures 2d and S3b). Closer inspection of the measured intensities in all four biological replicates revealed significantly different and clearly separated amounts of BRD2 and BRD4 proteins (Figure S4a). These data were substantiated by multiple quantified peptides per protein group (Figure S4b,c). Moreover, we confirmed that MZ1‐induced BRD4 ubiquitination was also robustly detected in the absence of the p97 inhibitor (Figure S4d and Table S2), whereas changes in BRD2 did not reach significance, presumably due to a lower number of detected peptides (Figure S4e,f). In sum, our data demonstrate the ability of the TUBE‐MS method to detect increases in small molecule‐induced protein ubiquitination.

### Inhibitors of DUBs and of p97 Alter the Cellular Polyubiquitome

**Figure 4:**
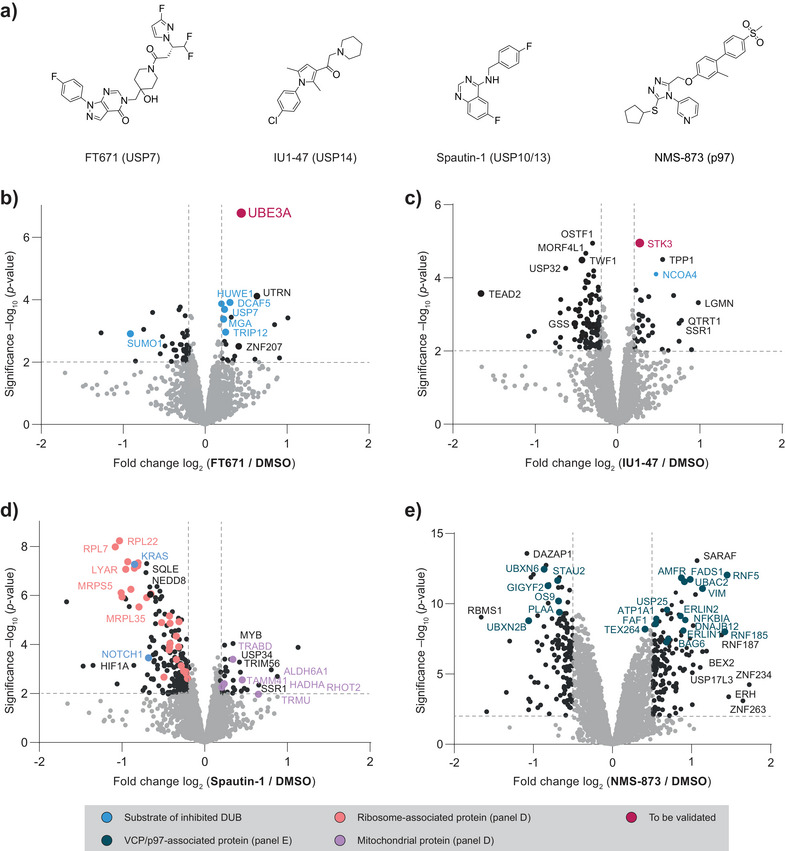
TUBE‐PD proteomics of cells treated with DUB and p97 inhibitors reveals proteome‐wide, compound‐induced changes in protein ubiquitination. a) Chemical structures of USP7 inhibitor FT671, USP14 inhibitor IU1‐47, USP10/13 inhibitor Spautin‐1 and p97 inhibitor NMS‐873. b–d) Volcano plots of enriched proteins after polyubiquitin PD from Jurkat cells being treated for 4 h with 1 µM FT671 (b), with 25 µM IU1‐47 (c) or with 10 µM Spautin‐1 (d), all compared to cells treated with DMSO. These data were recorded from samples without proteasome inhibition. e) Volcano plot of enriched proteins after polyubiquitin PD from Jurkat cells being treated for 5 h with 5 µM NMS‐873 and 0.5 µM CFZ compared to 0.5 µM CFZ. Proteins are colored as indicated in the legend. Proteomics data were recorded in quadruplicate from independently treated cell samples and were analyzed using FragPipe as described in the methods section.

These results motivated us to further explore the application scope of the assay. Since proteasomal inhibition could limit the assay's sensitivity due to accumulating ubiquitination on proteins with low to medium half‐life, we next assessed the workflow performance in the absence of the proteasomal inhibitor CFZ. We focused on deubiquitinase inhibitors as another class of ubiquitination‐altering compounds. We selected three compounds targeting members of the USP family of DUBs (Figure 4a): FT671^[^ ^36^ ^]^ is a highly potent, specific, and structurally characterized inhibitor of USP7, with sub‐µM potency and demonstrated cellular target engagement. IU1‐47^[^ ^37^ ^]^ represents a similarly established and characterized inhibitor of USP14, whereas Spautin‐1 is a reported inhibitor of USP10 and USP13, which was investigated in the context of melanoma cell growth inhibition.^[^ ^38^ ^]^ Furthermore, we analyzed the effect of p97 inhibition on the cellular ubiquitome using the highly potent p97 inhibitor NMS‐873.^[^ ^39^ ^]^ As p97 and the proteasome frequently cooperate in the turnover of ubiquitinated substrates, we investigated NMS‐873 in the presence of CFZ.

We treated Jurkat cells with an intact proteasome with the DUB inhibitors in four replicates and recorded data in a TMTpro 16plex experiment, which enabled identification and quantification of 4544 proteins (Table S3). Inhibition of USP7 by FT671 led to the detection of increased ubiquitination on several known USP7 substrates (Figure 4b). These include the PRC component MGA and the E3 ligases TRIP12, HUWE1, and DCAF5.^[^ ^40^, ^41^ ^]^ In addition, we also observed increased ubiquitination on USP7 itself, in line with reports on auto‐deubiquitination.^[^ ^27^ ^]^ Strikingly, the most significant hit was the E3 ligase UBE3A (also termed E6AP), which is the founding member of the ‘**H**omologous to the **E**6AP **C**arboxyl **T**erminus' (HECT) E3 ligase family. UBE3A has been extensively investigated due to its ability to ubiquitinate the p53 tumor suppressor upon human papillomavirus infection^[^ ^42^ ^]^ and due to its clinical significance with mutations causing Angelman syndrome and Prader‐Willi syndrome. However, it has not previously been reported as a USP7 substrate.

Treatment of cells with IU1‐47 and Spautin‐1 also led to changes in the polyubiquitination state of numerous proteins (Figure 4c,d), which include previously annotated substrates. For USP14, these include the STK3 kinase and the nuclear receptor coactivator 4 (NCOA4)^[^ ^43^ ^]^ and for USP10 the NOTCH1 receptor.^[^ ^44^ ^]^ Furthermore, our observations revealed the enrichment of several mitochondrial proteins in response to Spautin‐1 treatment, which is consistent with a recent report that established Spautin‐1 as an inducer of ubiquitination‐driven mitophagy.^[^ ^45^ ^]^ We also observed a decreased presence of 29 ribosomal proteins upon Spautin‐1 treatment, comprising components of both the small and the large ribosomal subunits as well as associated ribosomal biogenesis factors.^[^ ^46^, ^47^ ^]^ These findings are in line with a role of USP10 in regulating the turnover of ribosomal proteins.^[^ ^47^ ^]^

The diminished ubiquitination of ribosomal proteins, NOTCH1, and KRAS indicates an augmented degradation of DUB targets. To corroborate this observation, we carried out the same analysis for samples with proteasome inhibition using CFZ (Figure S5a–e). With this pretreatment of cells, we did not observe ribosomal proteins being significantly reduced in their ubiquitination compared to the data shown in Figure 4d, indicating a degradative nature of their polyubiquitination (Figure S5e). To comprehensively characterize the effect of proteasome inhibition on the cellular polyubiquitome, we treated cells with CFZ for 1 h and compared their polyubiquitome to cells with intact proteasome by TUBE‐MS (Figure S5c,d). This experiment revealed a large increase in enriched Ub (Figure S5a,b), which is consistent with significant changes in the polyubiquitination of many proteins (Figure S5c,d). This is in line with multiple previous studies that characterized the broad impact of proteasome inhibition on both protein levels as well as ubiquitination states.^[^ ^28^, ^48^, ^49^, ^50^ ^]^ Together, these data emphasize the broad utility of TUBE‐MS to report on both broad (Figure S5c,d) and specific (Figure S5e) changes in protein polyubiquitination.

Furthermore, we analyzed the effects of p97 inhibition and found many proteins changed by NMS‐873 (Figure 4e). Within this list, we identified several literature‐reported interactors of p97 as well as proteins involved in the p97‐regulated ERAD pathway.^[^ ^51^, ^52^, ^53^ ^]^

Notably, we observed all small molecule‐mediated effects already after four (DUB inhibitors) to 5 h (p97 inhibitor) of cell treatment, which stresses the assay's capability to detect emerging changes in target ubiquitination patterns upon acute perturbation. This also highlights the potential to investigate compounds such as NMS‐873, which become cytotoxic at longer treatment times and whose targets are thus challenging to study.^[^ ^39^ ^]^

### FT671 induces Non‐degradative Ubiquitination on the UBE3A E3 Ligase

Elevated signals upon DUB inhibition point towards increased protein ubiquitination; however, this effect could also be caused by secondary effects or higher protein levels despite the short treatment time. We thus conducted validation experiments on the two proteins STK3 and UBE3A, whose enrichment was detected with high statistical significance upon USP14 and USP7 inhibition, respectively, and which were not previously reported as substrates of these DUBs. Analysis of whole cell lysates of Jurkat cells by immunoblotting demonstrated that IU1‐47 caused a more than two‐fold increase in STK3 levels (Figure S6a,b), which suggests that the enhanced ubiquitination of the protein is derived from higher global STK3 levels, leading to proportionally enhanced ubiquitination. STK3 represents an upstream effector within the Hippo pathway, which itself is regulated by USP14 activity through a TAZ‐dependent feedback loop.^[^ ^54^ ^]^ As such, changes of STK3 protein levels upon USP14 inhibition are conceivable even without STK3 being directly deubiquitinated by USP14, and these data emphasize the need for validation.

**Figure 5:**
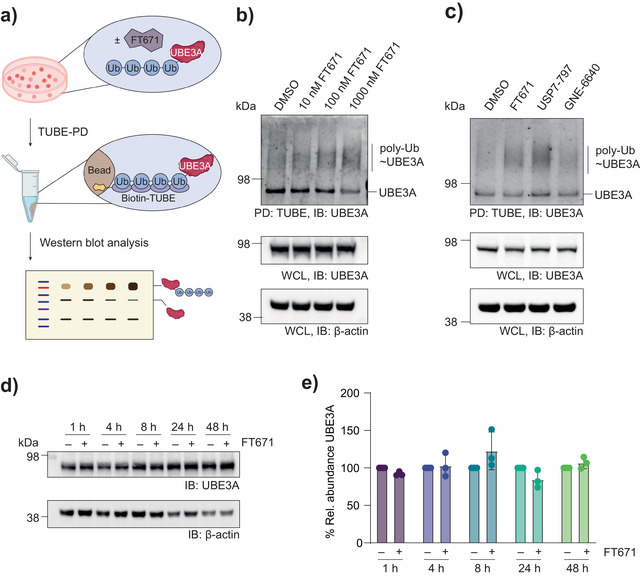
FT671 induces non‐degradative ubiquitination of UBE3A. a) Schematic of the experimental workflow used for proteomics validation. A TUBE‐PD from Jurkat cells, treated with FT671 or DMSO for 4 h, was analyzed by immunoblot for UBE3A. b) Western blot of enriched proteins after polyubiquitin PD from Jurkat cells being treated with indicated concentrations of FT671 or DMSO. Note the enhanced polyubiquitination (poly‐Ub) smear of UBE3A upon compound treatment. PD, pull down. WCL, whole cell lysate. c) Western blot of enriched proteins after polyubiquitin PD from Jurkat cells being treated with USP7 inhibitors FT671 (1 µM), USP7‐797 (0.5 µM), GNE‐6640 (5 µM) or DMSO. Please note that the strength of the effect correlates with the inhibitory potency of the compounds on recombinant USP7 (FT671: 52 nM, USP7‐797: 0.44 nM, GNE‐6640: 750 nM). d) Western blot analysis of Jurkat cells treated with FT671 (1 µM) for 1 to 48 h. e) Densitometric quantification of results shown in panel d. Bar graphs show mean ± s.d. (*N*=3) with individual replicates shown as dots. Treatment with FT671 does not change global UBE3A abundance, demonstrating non‐degradative ubiquitination of UBE3A. Uncropped versions of all blots are shown in the Supporting Information.

Lastly, we turned our attention to UBE3A (Figure 4b). To validate UBE3A ubiquitination upon USP7 inhibition, we performed immunoblotting of TUBE‐PD fractions (Figure 5a). We observed an enrichment of ubiquitinated UBE3A in a FT671‐ and dose‐dependent manner (Figure 5b). To corroborate this finding as a USP7‐mediated effect and to exclude potential off‐target effects, we performed the same experiments with the two structurally distinct USP7 inhibitors USP7‐797^[^ ^55^ ^]^ and GNE‐6640^[^ ^56^ ^]^ (Figure 5c). We observed enhanced polyubiquitination independent of the inhibitor scaffold, which suggests a physiological connection between USP7 and UBE3A. This finding is relevant in view of recent work that demonstrated antagonistic regulation of ubiquitination of the E3 ligase XIAP by USP7 and UBE3A.^[^ ^57^ ^]^ Although this work did not show an interaction between USP7 and UBE3A, our results suggest how their common substrate XIAP could facilitate effects of USP7 activity on UBE3A. Importantly, we did not observe any changes in overall UBE3A abundance upon Jurkat cell treatment with FT671 up to 48 h (Figure 5d,e). Taken together, these results demonstrate the induction of non‐degradative ubiquitination on UBE3A upon inhibition of USP7. More broadly, these data show that the TUBE‐MS method can detect small‐molecule‐induced protein ubiquitination and that it is complementary to other methods through its ability to report on non‐degradative polyubiquitin modifications.

## Discussion

We here report a method to map small‐molecule‐induced changes in protein polyubiquitination through mass spectrometry. We demonstrate that TUBE‐MS is capable of reporting on PROTAC‐mediated degradative ubiquitination (Figure 3) as well as of capturing broad changes in protein polyubiquitination induced by other bioactive molecules irrespective of proteasomal inhibition (Figure 4). We anticipate that this workflow will be readily adopted by the chemical biology community for the characterization of bioactive compounds.

We utilized TUBEs^[^ ^22^ ^]^ for polyubiquitin enrichment and enhanced the workflow towards sensitive detection of ubiquitinated proteins via PDs coupled to LC‐MS/MS. Notably, previous approaches have demonstrated the potential of such a coupling,^[^ ^24^, ^58^ ^]^ including examples on yeast,^[^ ^26^, ^59^ ^]^ plant,^[^ ^60^ ^]^ and malaria parasite‐infected blood samples.^[^ ^61^ ^]^ However, earlier approaches focused on pure detection^[^ ^24^ ^]^ of polyubiquitinated proteins rather than quantitative comparison of samples. Moreover, most previous approaches used gel‐based proteomics to circumvent the spectral suppression by large amounts of the TUBE protein in samples, thus reaching only up to about 1000 quantified proteins, even in recent approaches.^[^ ^26^, ^61^, ^62^ ^]^ Importantly, this workflow was proposed for the characterization of PROTAC‐mediated protein ubiquitination,^[^ ^63^ ^]^ but data exemplifying its potential has not been published. To solve these issues, the method reported here is based on the separation of polyubiquitinated proteins from the TUBE reagent through acidic elution (Figure 2). We further combined SP3‐based sample preparation and TMT‐based mass spectrometric quantification to achieve high sensitivity, resulting in the robust quantification of more than 6000 polyubiquitinated proteins. Taken together, our data demonstrate the advance this method provides over previous work.

Considering the exceptionally low stoichiometry of ubiquitination sites,^[^ ^16^, ^21^ ^]^ the low proportion of polyubiquitin chains within the ubiquitin pool,^[^ ^64^ ^]^ and their high turnover, highly specific and efficient enrichment of polyubiquitin is essential. We here used the 4xUBA^UBQLN1^ TUBE protein^[^ ^22^ ^]^ in combination with semi‐denaturing lysis conditions^[^ ^29^ ^]^ in order to suppress the co‐purification of non‐ubiquitinated proteins and the Ub interactome which exist in cells to a large excess. Although biotinylated TUBE reagents are commercially available, a method for their preparation was not reported. We here describe the facile and cost‐effective preparation of a biotinylated TUBE reagent enabling swift and modular preparation of TUBE‐bead‐assemblies. The site‐specific modification complements non‐specific^[^ ^65^ ^]^ or cross‐linking‐based^[^ ^61^ ^]^ immobilization of polyubiquitin enrichment reagents.

The critical role of protein polyubiquitination is also reflected in the rapid development of the field of targeted protein degradation as well as multiple other small molecule‐based modalities.^[^ ^16^, ^66^ ^]^ These advances stress the demand for robust methods to facilitate a proteome‐wide monitoring of compound‐induced changes in protein polyubiquitination. We are thus confident that the TUBE‐MS method represents a valuable addition to the repertoire of assays suitable for the discovery of bioactive compounds interfering with ubiquitination (Figure 1).

TUBE‐MS synergizes with other recently reported proteomics approaches to investigate PROTACS, DUB inhibitors and molecular glue degraders.^[^ ^66^ ^]^ These include i) the quantification of degrader‐induced protein decay by pulsed‐SILAC coupled to translatome labeling,^[^ ^18^ ^]^ ii) detection of deubiquitinase substrates by either broad or specific DUB inhibition,^[^ ^41^, ^67^ ^]^ and iii) proximity‐based labeling to identify E3 ligase substrates.^[^ ^68^, ^69^ ^]^ In addition, there has been notable progress in the application of data‐independent acquisition mass spectrometry in combination with diGly proteomics,^[^ ^19^ ^]^ including case studies on USP7^[^ ^27^ ^]^ and on the mitochondrial DUB USP30.^[^ ^70^ ^]^

It is important to highlight how TUBE‐MS is complementary to these other approaches: Firstly, diGly proteomics detects individual peptides with ubiquitin/NEDD8/ISG15 remnants, irrespective of the ubiquitin chain architecture.^[^ ^19^, ^21^ ^]^ In contrast, protein quantification through TUBE‐MS relies on multiple peptides per protein and, through the avidity of the reagent, specifically enriches proteins modified with polyubiquitin chains. Secondly, in contrast to approaches involving cellular expression of TUBEs^[^ ^25^ ^]^ or of epitope‐tagged ubiquitin,^[^ ^71^ ^]^ the here reported method enriches endogenous, untagged ubiquitin chains and thus enables assessment of small molecule‐mediated effects in an otherwise unperturbed background. Thirdly, multiple methods focus on changes in protein abundance as an indirect proxy for degradative polyubiquitin modification.^[^ ^17^, ^41^ ^]^ TUBE‐MS, however, focuses on the detection of protein ubiquitination, thus excludes secondary (translational) effects as the cause of a change in global protein abundance and covers nondegradative polyubiquitin modification as well. This is exemplified through the non‐degradative ubiquitination on the E3 ligase UBE3A induced by USP7 inhibition (Figure 5). Future work will need to establish whether USP7 interacts with UBE3A directly or through common binding partners. Moreover, the functional consequence of this non‐degradative modification on UBE3A will need to be evaluated.

## Conclusion

In sum, we contribute a robust readout of polyubiquitination and the complementary ability to detect non‐degradative polyubiquitin modifications. We are convinced that the TUBE‐MS method will find widespread use for the identification and characterization of Ub‐dependent bioactivity in small molecules.

## Supporting Information

Supporting Information for this manuscript includes Methods, Figures S1–S6, Uncropped gels and blots, and Supporting References,^[^ ^72^, ^73^, ^74^, ^75^, ^76^, ^77^, ^78^, ^79^, ^80^ ^]^ and is provided as a pdf file. Tables S1–S4 are provided as xlsx files.

## Author Contributions

S.F. performed assays, cellular experiments, validation of assay hits, protein expression, and biotinylation, and analyzed proteomics data. K.G. cloned the TUBE construct and supported assays. F.K., M.K., and P.J. acquired and analyzed proteomics data, oversaw proteomics measurements, and advised on mass spectrometry. M.G. and H.W. supervised the project. S.F., H.W., and M.G. wrote the manuscript with contributions by all authors.

## Conflict of Interests

The authors declare no conflict of interest.

## Supporting information



Supporting Information

Supporting Information

Supporting Information

Supporting Information

Supporting Information

## Data Availability

Proteomics results are supplied as Table S1 (data shown in Figure 3c), Table S2 (data shown in Figure S4d), Table S3 (data shown in Figure 4b–e), and Table S4 (data shown in Figure S5c–e). Proteomics raw data are available via ProteomeXchange (ID: PXD057541 and PXD062845). Uncropped images of gels and blots are available in the Supporting Information.

## References

[1] I. Dikic , B. A. Schulman , Nat. Rev. Mol. Cell Biol. 2023, 24, 273–287. 36284179 10.1038/s41580-022-00543-1 PMC9595094

[2] K. N. Swatek , D. Komander , Cell Res. 2016, 26, 399–422. 27012465 10.1038/cr.2016.39 PMC4822133

[3] M. L. Mendes , M. R. Fougeras , G. Dittmar , J. Proteomics 2020, 215, 103634. 31918034 10.1016/j.jprot.2020.103634

[4] J. Lutz , E. Hollmuller , M. Scheffner , A. Marx , F. Stengel , Angew. Chem. Int. Ed. Engl. 2020, 59, 12371–12375. 32301549 10.1002/anie.202003058 PMC7384046

[5] Y. K. Satija , A. Bhardwaj , S. Das , Int. J. Cancer 2013, 133, 2759–2768. 23436247 10.1002/ijc.28129

[6] S. M. Lange , L. A. Armstrong , Y. Kulathu , Mol. Cell 2022, 82, 15–29. 34813758 10.1016/j.molcel.2021.10.027

[7] D. Conole , F. Cao , C. W. Am Ende , L. Xue , S. Kantesaria , D. Kang , J. Jin , D. Owen , L. Lohr , M. Schenone , J. D. Majmudar , E. W. Tate , Angew. Chem. Int. Ed. Engl. 2023, 62, e202311190. 37779326 10.1002/anie.202311190

[8] J. Kronke , N. D. Udeshi , A. Narla , P. Grauman , S. N. Hurst , M. McConkey , T. Svinkina , D. Heckl , E. Comer , X. Li , C. Ciarlo , E. Hartman , N. Munshi , M. Schenone , S. L. Schreiber , S. A. Carr , B. L. Ebert , Science 2014, 343, 301–305. 24292625 10.1126/science.1244851 PMC4077049

[9] D. P. Bondeson , C. M. Crews , Annu. Rev. Pharmacol. Toxicol. 2017, 57, 107–123. 27732798 10.1146/annurev-pharmtox-010715-103507 PMC5586045

[10] K. M. Sakamoto , K. B. Kim , A. Kumagai , F. Mercurio , C. M. Crews , R. J. Deshaies , Proc. Natl. Acad. Sci. U. S. A. 2001, 98, 8554–8559. 11438690 10.1073/pnas.141230798 PMC37474

[11] M. Winzker , A. Friese , U. Koch , P. Janning , S. Ziegler , H. Waldmann , Angew. Chem. Int. Ed. Engl. 2020, 59, 5595–5601. 31829492 10.1002/anie.201913904 PMC7154537

[12] Y. Pei , J. Fu , Y. Shi , M. Zhang , G. Luo , X. Luo , N. Song , T. Mi , Y. Yang , J. Li , Y. Zhou , B. Zhou , Angew. Chem. Int. Ed. Engl. 2022, 61, e202204395. 35691827 10.1002/anie.202204395

[13] M. Teng , J. Jiang , Z. He , N. P. Kwiatkowski , K. A. Donovan , C. E. Mills , C. Victor , J. M. Hatcher , E. S. Fischer , P. K. Sorger , T. Zhang , N. S. Gray , Angew. Chem. Int. Ed. Engl. 2020, 59, 13865–13870. 32415712 10.1002/anie.202004087 PMC7703486

[14] M. Therapeutics , Mission Therapeutics, 2023. https://missiontherapeutics.com/mission‐therapeutics‐announces‐us‐fda‐approval‐to‐initiate‐phase‐ii‐clinical‐trial‐of‐its‐lead‐asset‐mtx652‐in‐acute‐kidney‐injury/

[15] L. Cadzow , J. Brenneman , E. Tobin , P. Sullivan , S. Nayak , J. A. Ali , S. Shenker , J. Griffith , M. McGuire , P. Grasberger , Y. Mishina , M. Murray , A. E. Dodson , H. Gannon , E. Krall , J. Hixon , E. Chipumuro , K. Sinkevicius , P. C. Gokhale , S. Ganapathy , U. A. Matulonis , J. F. Liu , A. Olaharski , D. Sangurdekar , H. Liu , J. Wilt , M. Schlabach , F. Stegmeier , A. A. Wylie , Cancer Res. 2024, 84, 3419–3434. 39402989 10.1158/0008-5472.CAN-24-0293 PMC11474170

[16] N. S. Scholes , C. Mayor‐Ruiz , G. E. Winter , Cell Chem. Biol. 2021, 28, 1048–1060. 33811812 10.1016/j.chembiol.2021.03.007

[17] C. Mayor‐Ruiz , S. Bauer , M. Brand , Z. Kozicka , M. Siklos , H. Imrichova , I. H. Kaltheuner , E. Hahn , K. Seiler , A. Koren , G. Petzold , M. Fellner , C. Bock , A. C. Muller , J. Zuber , M. Geyer , N. H. Thoma , S. Kubicek , G. E. Winter , Nat. Chem. Biol. 2020, 16, 1199–1207. 32747809 10.1038/s41589-020-0594-x PMC7116640

[18] M. Jochem , A. Schrempf , L. M. Wagner , D. Segal , J. Cisneros , A. Ng , G. E. Winter , J. Krijgsveld , Cell Chem. Biol. 2025, 32, 192–200. 39536762 10.1016/j.chembiol.2024.10.007

[19] F. M. Hansen , M. C. Tanzer , F. Bruning , I. Bludau , C. Stafford , B. A. Schulman , M. S. Robles , O. Karayel , M. Mann , Nat. Commun. 2021, 12, 254. 33431886 10.1038/s41467-020-20509-1 PMC7801436

[20] H. Wolf‐Levy , A. Javitt , A. Eisenberg‐Lerner , A. Kacen , A. Ulman , D. Sheban , B. Dassa , V. Fishbain‐Yoskovitz , C. Carmona‐Rivera , M. P. Kramer , N. Nudel , I. Regev , L. Zahavi , D. Elinger , M. J. Kaplan , D. Morgenstern , Y. Levin , Y. Merbl , Nat. Biotechnol. 2018, 36, 1110–1116. 10.1038/nbt.4279 PMC8897557 30346940

[21] G. Prus , S. Satpathy , B. T. Weinert , T. Narita , C. Choudhary , Cell 2024, 187, 2875 ‐2892. 38626770 10.1016/j.cell.2024.03.024 PMC11136510

[22] R. Hjerpe , F. Aillet , F. Lopitz‐Otsoa , V. Lang , P. England , M. S. Rodriguez , EMBO Rep. 2009, 10, 1250–1258. 19798103 10.1038/embor.2009.192 PMC2775171

[23] J. J. Sims , F. Scavone , E. M. Cooper , L. A. Kane , R. J. Youle , J. D. Boeke , R. E. Cohen , Nat. Methods 2012, 9, 303–309. 22306808 10.1038/nmeth.1888 PMC3438894

[24] Y. Shi , D. W. Chan , S. Y. Jung , A. Malovannaya , Y. Wang , J. Qin , Mol. Cell. Proteomics 2011, 10, M110002089. 10.1074/mcp.M110.002089 PMC3098580 20972266

[25] Y. Yoshida , Y. Saeki , A. Murakami , J. Kawawaki , H. Tsuchiya , H. Yoshihara , M. Shindo , K. Tanaka , Proc Natl Acad Sci U S A 2015, 112, 4630–4635. 25827227 10.1073/pnas.1422313112 PMC4403176

[26] Y. Gao , Y. Li , C. Zhang , M. Zhao , C. Deng , Q. Lan , Z. Liu , N. Su , J. Wang , F. Xu , Y. Xu , L. Ping , L. Chang , H. Gao , J. Wu , Y. Xue , Z. Deng , J. Peng , P. Xu , Mol. Cell. Proteomics 2016, 15, 1381–1396. 27037361 10.1074/mcp.O115.051839 PMC4824862

[27] M. Steger , V. Demichev , M. Backman , U. Ohmayer , P. Ihmor , S. Muller , M. Ralser , H. Daub , Nat. Commun. 2021, 12, 5399. 34518535 10.1038/s41467-021-25454-1 PMC8438043

[28] B. A. Maxwell , Y. Gwon , A. Mishra , J. Peng , H. Nakamura , K. Zhang , H. J. Kim , J. P. Taylor , Science 2021, 372, eabc3593. 34739326 10.1126/science.abc3593 PMC8574219

[29] M. Zhang , J. M. Berk , A. B. Mehrtash , J. Kanyo , M. Hochstrasser , PLoS Biol. 2022, 20, e3001501. 35771886 10.1371/journal.pbio.3001501 PMC9278747

[30] M. Fairhead , M. Howarth , Site‐Specific Protein Labeling: Methods and Protocols (Eds.: A. Gautier , M. J. Hinner ), Springer New York 2015, pp. 171–184.

[31] E. B. Dammer , C. H. Na , P. Xu , N. T. Seyfried , D. M. Duong , D. Cheng , M. Gearing , H. Rees , J. J. Lah , A. I. Levey , J. Rush , J. Peng , J. Biol. Chem. 2011, 286, 10457–10465. 21278249 10.1074/jbc.M110.149633 PMC3060499

[32] K. N. Swatek , J. L. Usher , A. F. Kueck , C. Gladkova , T. E. T. Mevissen , J. N. Pruneda , T. Skern , D. Komander , Nature 2019, 572, 533–537. 31413367 10.1038/s41586-019-1482-y PMC6823057

[33] M. Zengerle , K. H. Chan , A. Ciulli , ACS Chem. Biol. 2015, 10, 1770–1777. 26035625 10.1021/acschembio.5b00216 PMC4548256

[34] K. Wendrich , K. Gallant , S. Recknagel , S. Petroulia , N. H. Kazi , J. A. Hane , S. Fuhrer , K. Bezstarosti , R. O'Dea , J. Demmers , M. Gersch , Nat. Chem. Biol. 2025, 21, 746–757. 39587316 10.1038/s41589-024-01777-0 PMC12037411

[35] C. S. Hughes , S. Moggridge , T. Muller , P. H. Sorensen , G. B. Morin , J. Krijgsveld , Nat. Protoc. 2019, 14, 68–85. 30464214 10.1038/s41596-018-0082-x

[36] A. P. Turnbull , S. Ioannidis , W. W. Krajewski , A. Pinto‐Fernandez , C. Heride , A. C. L. Martin , L. M. Tonkin , E. C. Townsend , S. M. Buker , D. R. Lancia , J. A. Caravella , A. V. Toms , T. M. Charlton , J. Lahdenranta , E. Wilker , B. C. Follows , N. J. Evans , L. Stead , C. Alli , V. V. Zarayskiy , A. C. Talbot , A. J. Buckmelter , M. Wang , C. L. McKinnon , F. Saab , J. F. McGouran , H. Century , M. Gersch , M. S. Pittman , C. G. Marshall , et al., Nature 2017, 550, 481–486. 29045389 10.1038/nature24451 PMC6029662

[37] B. H. Lee , M. J. Lee , S. Park , D. C. Oh , S. Elsasser , P. C. Chen , C. Gartner , N. Dimova , J. Hanna , S. P. Gygi , S. M. Wilson , R. W. King , D. Finley , Nature 2010, 467, 179–184. 20829789 10.1038/nature09299 PMC2939003

[38] J. Guo , J. Zhang , L. Liang , N. Liu , M. Qi , S. Zhao , J. Su , J. Liu , C. Peng , X. Chen , H. Liu , J. Cell. Mol. Med. 2020, 24, 4324–4340. 32129945 10.1111/jcmm.15093 PMC7171391

[39] P. Magnaghi , R. D'Alessio , B. Valsasina , N. Avanzi , S. Rizzi , D. Asa , F. Gasparri , L. Cozzi , U. Cucchi , C. Orrenius , P. Polucci , D. Ballinari , C. Perrera , A. Leone , G. Cervi , E. Casale , Y. Xiao , C. Wong , D. J. Anderson , A. Galvani , D. Donati , T. O'Brien , P. K. Jackson , A. Isacchi , Nat. Chem. Biol. 2013, 9, 548–556. 23892893 10.1038/nchembio.1313

[40] M. E. Sowa , E. J. Bennett , S. P. Gygi , J. W. Harper , Cell 2009, 138, 389–403. 19615732 10.1016/j.cell.2009.04.042 PMC2716422

[41] J. W. Bushman , K. A. Donovan , N. J. Schauer , X. Liu , W. Hu , A. C. Varca , S. J. Buhrlage , E. S. Fischer , Cell Chem. Biol. 2021, 28, 78–87. 33007217 10.1016/j.chembiol.2020.09.005 PMC7855594

[42] M. Scheffner , B. A. Werness , J. M. Huibregtse , A. J. Levine , P. M. Howley , Cell 1990, 63, 1129–1136. 2175676 10.1016/0092-8674(90)90409-8

[43] C. Li , G. Sun , B. Chen , L. Xu , Y. Ye , J. He , Z. Bao , P. Zhao , Z. Miao , L. Zhao , J. Hu , Y. You , N. Liu , H. Chao , J. Ji , Pharmacol Res 2021, 174, 105933. 34634471 10.1016/j.phrs.2021.105933

[44] R. Lim , T. Sugino , H. Nolte , J. Andrade , B. Zimmermann , C. Shi , A. Doddaballapur , Y. T. Ong , K. Wilhelm , J. W. D. Fasse , A. Ernst , M. Kaulich , K. Husnjak , T. Boettger , S. Guenther , T. Braun , M. Kruger , R. Benedito , I. Dikic , M. Potente , Science 2019, 364, 188–193. 30975888 10.1126/science.aat0778

[45] J. Yi , H. L. Wang , G. Lu , H. Zhang , L. Wang , Z. Y. Li , L. Wang , Y. Wu , D. Xia , E. F. Fang , H. M. Shen , Autophagy 2024, 20, 2655–2676. 39051473 10.1080/15548627.2024.2383145 PMC11587853

[46] A. R. Lopez , M. H. Jorgensen , J. F. Havelund , F. S. Arendrup , S. P. Kolapalli , T. M. Nielsen , E. Pais , C. J. Beese , A. Abdul‐Al , A. C. Vind , J. Bartek , S. Bekker‐Jensen , M. Montes , P. Galanos , N. Faergeman , L. Happonen , L. B. Frankel , Cell Rep. 2023, 42, 113381. 37930887 10.1016/j.celrep.2023.113381

[47] C. Meyer , A. Garzia , P. Morozov , H. Molina , T. Tuschl , Mol. Cell 2020, 77, 1193‐1205. 31981475 10.1016/j.molcel.2019.12.024

[48] F. Trulsson , V. Akimov , M. Robu , N. van Overbeek , D. A. P. Berrocal , R. G. Shah , J. Cox , G. M. Shah , B. Blagoev , A. C. O. Vertegaal , Nat. Commun. 2022, 13, 2736. 35585066 10.1038/s41467-022-30376-7 PMC9117253

[49] J. Currie , V. Manda , S. K. Robinson , C. Lai , V. Agnihotri , V. Hidalgo , R. W. Ludwig , K. Zhang , J. Pavelka , Z. V. Wang , J. W. Rhee , M. P. Y. Lam , E. Lau , Nat. Commun. 2024, 15, 2207. 38467653 10.1038/s41467-024-46600-5 PMC10928085

[50] T. R. Porras‐Yakushi , J. M. Reitsma , M. J. Sweredoski , R. J. Deshaies , S. Hess , J. Proteomics 2021, 241, 104197. 33848640 10.1016/j.jprot.2021.104197

[51] P. Hanzelmann , H. Schindelin , J. Biol. Chem. 2011, 286, 38679–38690. 21914798 10.1074/jbc.M111.274506 PMC3207442

[52] M. J. Brody , D. Vanhoutte , C. V. Bakshi , R. Liu , R. N. Correll , M. A. Sargent , J. D. Molkentin , J. Biol. Chem. 2019, 294, 8918–8929. 31006653 10.1074/jbc.RA119.007585 PMC6552413

[53] M. H. Lehner , J. Walker , K. Temcinaite , A. Herlihy , M. Taschner , A. C. Berger , A. H. Corbett , A. B. Dirac Svejstrup , J. Q. Svejstrup , Cell Rep. 2022, 41, 111536. 36288698 10.1016/j.celrep.2022.111536 PMC9638028

[54] C. Zhao , J. Gong , Y. Bai , T. Yin , M. Zhou , S. Pan , Y. Liu , Y. Gao , Z. Zhang , Y. Shi , F. Zhu , H. Zhang , M. Wang , R. Qin , Cell Death Differ. 2023, 30, 1–15. 35906484 10.1038/s41418-022-01040-w PMC9883464

[55] P. R. Leger , D. X. Hu , B. Biannic , M. Bui , X. Han , E. Karbarz , J. Maung , A. Okano , M. Osipov , G. M. Shibuya , K. Young , C. Higgs , B. Abraham , D. Bradford , C. Cho , C. Colas , S. Jacobson , Y. M. Ohol , D. Pookot , P. Rana , J. Sanchez , N. Shah , M. Sun , S. Wong , D. G. Brockstedt , P. D. Kassner , J. B. Schwarz , D. J. Wustrow , J. Med. Chem. 2020, 63, 5398–5420. 32302140 10.1021/acs.jmedchem.0c00245

[56] L. Kategaya , P. Di Lello , L. Rouge , R. Pastor , K. R. Clark , J. Drummond , T. Kleinheinz , E. Lin , J. P. Upton , S. Prakash , J. Heideker , M. McCleland , M. S. Ritorto , D. R. Alessi , M. Trost , T. W. Bainbridge , M. C. M. Kwok , T. P. Ma , Z. Stiffler , B. Brasher , Y. Tang , P. Jaishankar , B. R. Hearn , A. R. Renslo , M. R. Arkin , F. Cohen , K. Yu , F. Peale , F. Gnad , M. T. Chang , et al., Nature 2017, 550, 534–538. 29045385 10.1038/nature24006

[57] H. Qiao , Y. Tian , Y. Huo , H. Y. Man , iScience 2022, 25, 104595. 35800757 10.1016/j.isci.2022.104595 PMC9253496

[58] F. Lopitz‐Otsoa , E. Rodriguez‐Suarez , F. Aillet , J. Casado‐Vela , V. Lang , R. Matthiesen , F. Elortza , M. S. Rodriguez , J. Proteomics 2012, 75, 2998–3014. 22178446 10.1016/j.jprot.2011.12.001

[59] G. M. Silva , D. Finley , C. Vogel , Nat. Struct. Mol. Biol. 2015, 22, 116–123. 25622294 10.1038/nsmb.2955 PMC4318705

[60] A. Johnson , G. Vert , Plant Physiol. 2016, 171, 1808–1820. 27208306 10.1104/pp.16.00619 PMC4936586

[61] L. Mata‐Cantero , M. Azkargorta , F. Aillet , W. Xolalpa , M. J. LaFuente , F. Elortza , A. S. Carvalho , J. Martin‐Plaza , R. Matthiesen , M. S. Rodriguez , J. Proteomics 2016, 139, 45–59. 26972027 10.1016/j.jprot.2016.03.004

[62] G. Quinet , W. Xolalpa , D. Reyes‐Garau , N. Profitos‐Peleja , M. Azkargorta , L. Ceccato , M. Gonzalez‐Santamarta , M. Marsal , J. Andilla , F. Aillet , F. Bosch , F. Elortza , P. Loza‐Alvarez , B. Sola , O. Coux , R. Matthiesen , G. Roue , M. S. Rodriguez , Cancers (Basel) 2022, 14, 923. 35205670 10.3390/cancers14040923 PMC8869867

[63] K. Kadimisetty , K. J. Sheets , P. H. Gross , M. J. Zerr , D. Ouazia , Methods Mol. Biol. 2021, 2365, 185–202. 34432245 10.1007/978-1-0716-1665-9_10

[64] S. E. Kaiser , B. E. Riley , T. A. Shaler , R. S. Trevino , C. H. Becker , H. Schulman , R. R. Kopito , Nat. Methods 2011, 8, 691–696. 21743460 10.1038/nmeth.1649 PMC3196335

[65] X. Cheng , Y. Wang , J. Liu , Y. Wu , Z. Zhang , H. Liu , L. Tian , L. Zhang , L. Chang , P. Xu , L. Zhang , Y. Li , Mol. Cell. Proteomics 2024, 23, 100852. 39362602 10.1016/j.mcpro.2024.100852 PMC11584597

[66] G. Sathe , G. P. Sapkota , Trends Pharmacol. Sci. 2023, 44, 786–801. 37778939 10.1016/j.tips.2023.08.007

[67] V. Rossio , J. A. Paulo , J. Chick , B. Brasher , S. P. Gygi , R. W. King , Cell Chem. Biol. 2021, 28, 487–502 33417828 10.1016/j.chembiol.2020.12.007 PMC8052291

[68] H. T. Huang , R. J. Lumpkin , R. W. Tsai , S. Su , X. Zhao , Y. Xiong , J. Chen , N. Mageed , K. A. Donovan , E. S. Fischer , W. R. Sellers , Nat. Chem. Biol. 2024, 20, 1227–1236. 38514884 10.1038/s41589-024-01590-9

[69] O. Barroso‐Gomila , L. Merino‐Cacho , V. Muratore , C. Perez , V. Taibi , E. Maspero , M. Azkargorta , I. Iloro , F. Trulsson , A. C. O. Vertegaal , U. Mayor , F. Elortza , S. Polo , R. Barrio , J. D. Sutherland , Nat. Commun. 2023, 14, 7656. 37996419 10.1038/s41467-023-43326-8 PMC10667490

[70] A. Damianou , H. B. L. Jones , A. Grigoriou , M. A. Akbor , E. Jenkins , P. D. Charles , I. Vendrell , S. Davis , B. M. Kessler , Cell Chem. Biol. 2025, 32, 736–751 40328249 10.1016/j.chembiol.2025.04.004

[71] K. Kliza , C. Taumer , I. Pinzuti , M. Franz‐Wachtel , S. Kunzelmann , B. Stieglitz , B. Macek , K. Husnjak , Nat. Methods 2017, 14, 504–512. 28319114 10.1038/nmeth.4228

[72] M. Schmidt , C. Grethe , S. Recknagel , G. M. Kipka , N. Klink , M. Gersch , Angew. Chem. Int. Ed. Engl. 2024, 63, e202318849. 38239128 10.1002/anie.202318849

[73] J. D. Holman , D. L. Tabb , P. Mallick , Curr. Protoc. Bioinformatics 2014, 46, 13.24.1–13.24.9. 10.1002/0471250953.bi1324s46 PMC4113728 24939128

[74] F. da Veiga Leprevost , S. E. Haynes , D. M. Avtonomov , H. Y. Chang , A. K. Shanmugam , D. Mellacheruvu , A. T. Kong , A. I. Nesvizhskii , Nat. Methods 2020, 17, 869–870. 32669682 10.1038/s41592-020-0912-y PMC7509848

[75] L. Kall , J. D. Canterbury , J. Weston , W. S. Noble , M. J. MacCoss , Nat. Methods 2007, 4, 923–925. 17952086 10.1038/nmeth1113

[76] A. T. Kong , F. V. Leprevost , D. M. Avtonomov , D. Mellacheruvu , A. I. Nesvizhskii , Nat. Methods 2017, 14, 513–520. 28394336 10.1038/nmeth.4256 PMC5409104

[77] A. I. Nesvizhskii , A. Keller , E. Kolker , R. Aebersold , Anal. Chem. 2003, 75, 4646–4658. 14632076 10.1021/ac0341261

[78] G. C. Teo , D. A. Polasky , F. Yu , A. I. Nesvizhskii , J. Proteome Res. 2021, 20, 498–505. 33332123 10.1021/acs.jproteome.0c00544 PMC8864561

[79] K. L. Yang , F. Yu , G. C. Teo , K. Li , V. Demichev , M. Ralser , A. I. Nesvizhskii , Nat. Commun. 2023, 14, 4539. 37500632 10.1038/s41467-023-40129-9 PMC10374903

[80] D. Kohler , M. Kaza , C. Pasi , T. Huang , M. Staniak , D. Mohandas , E. Sabido , M. Choi , O. Vitek , J. Proteome Res. 2023, 22, 551–556. 36622173 10.1021/acs.jproteome.2c00603

