# Supplementary material for: Small Molecule‐Induced Alterations of Protein Polyubiquitination Revealed by Mass‐Spectrometric Ubiquitome Analysis
Source: Angew Chem Int Ed Engl. 2025 Jun 26;64(32):e202508916. doi: 10.1002/anie.202508916 (PMC12322627; doi:10.1002/anie.202508916)
Supplement: Supplementary file 1 — Supporting Information [file ANIE-64-e202508916-s003.pdf]

Supporting Information

Small Molecule-Induced Alterations of Protein Polyubiquitination Revealed by Mass-Spectrometric Ubiquitome Analysis

Siska Führer[a,b,c], Kai Gallant[a,c], Farnusch Kaschani[d], Markus Kaiser[d], Petra Janning[b], Herbert Waldmann*[a,b,c] and Malte Gersch*[a,c]

[a] Max Planck Institute of Molecular Physiology, Chemical Genomics Centre, Dortmund, Germany.

[b] Max Planck Institute of Molecular Physiology, Department of Chemical Biology, Dortmund, Germany.

[c] TU Dortmund University, Department of Chemistry and Chemical Biology, Dortmund, Germany.

[d] University of Duisburg-Essen, Faculty of Biology, Analytics Core Facility Essen (ACE), ZMB, Essen, Germany.

*Correspondence: herbert.waldmann@mpi-dortmund.mpg.de; malte.gersch@mpi-dortmund.mpg.de

Table of Contents

Methods .... page 2

Figures S1-S6 .... page 11

Uncropped gels and blots .... page 18

Supporting References .... page 30

Tables S1-S4 are supplied as separate files.

Methods

Expression of Avi-tagged TUBE and BirA

A sequence encoding the 4xUBA^{UBQLN1} TUBE[1] protein with an N-terminal Avi-tag was cloned into a pOPIN-E vector using the In-Fusion HD Cloning Kit (Takara Clonetech). BirA cloned in a pET21a vector was a kind gift from Mariann Bienz (MRC LMB, Cambridge, UK).

Plasmids were transformed into *E. coli* BL21(DE3). Bacteria were grown in 2xYT medium at 37 °C until an optical density of 0.8 to 1.2 was reached, upon which protein expression was induced by addition of IPTG to a final concentration of 0.5 mM. Cells were incubated overnight at 18 °C and afterwards harvested by centrifugation at 4 000 xg for 10 min. Cell pellets were stored at -80 °C until further use. Bacteria were lysed in ice-cold buffer (50 mM H_2NaPO_4, 300 mM NaCl, 20 mM imidazole, 4 mM beta-mercaptoethanol, pH 8.0), to which were added lysozyme and DNAse. The lysate was sonicated, cleared by centrifugation at 22 000 xg at 4 °C for 30 to 40 min and afterwards filtered with 0.45 µm syringe filters.

Protein purification was carried out on an Äkta Pure (Cytiva). His-tagged proteins were isolated using a 5 mL HisTrap column and eluted with high imidazole buffer (50 mM H_2NaPO_4, 300 mM NaCl, 500 mM imidazole, 4 mM beta-mercaptoethanol, pH 8.0). BirA was further purified by size exclusion chromatography in PBS. Avi-TUBE eluates were dialyzed into low salt buffer (25 mM Tris, 50 mM NaCl, 4 mM DTT, pH 8.5) and further purified by ion exchange chromatography using a ResQ column with gradient elution to high salt buffer (25 mM Tris, 500 mM NaCl, 4 mM DTT, pH 8.5). Protein purity and identity were assessed using SDS-PAGE and intact protein LC-MS as reported previously[2]. Protein concentration was determined either by Nanodrop (Thermo Fisher Scientific), microBCA protein assay (Thermo Fisher Scientific) or DC assay (Bio-Rad).

Biotinylation of Avi-TUBE

To Avi-TUBE protein (0.09 mM, 1 equiv.) in ResQ high salt buffer were added $MgCl_2$ (4.5 mM, 50 equiv.), BirA (0.005 mM, 0.06 equiv.), Biotin (1.0 mM, 11 equiv.) and ATP (1.8 mM, 20 equiv.). The solution was gently mixed overnight at 4 °C before complete conversion was assessed by intact protein LC-MS measurements. The reaction mixture was then dialyzed overnight into ResQ low salt buffer, and the Biotin-TUBE reagent was subsequently purified by anion exchange chromatography as described above.

In vitro pulldown of tetraUb chains

360 µL of streptavidin magnetic beads (NEB, #S1421S) were suspended in 0.9 mL dilution buffer (10 mM Tris, 150 mM NaCl, 1 mM EDTA, pH 7.5), pulled down with a magnet, washed twice with dilution buffer and resuspended in the same volume. The equivalent of 40 µL of bead suspension (160 µg) were transferred into fresh tubes and pulled down with a magnet. The supernatant was removed and 80 µL of Biotin-TUBE solution (64 pmol, 1 equiv.) or biocytin control (64 pmol, 1 equiv.) were added onto the beads. Afterwards, 80 µL of dilution buffer were added, beads were incubated for 1 h and then washed, before 80 µL of differently linked tetraUb chains (M1, K63, K48, 48 pmol, 0.75 equiv.) in solution and 80 µL of dilution buffer were added. Tubes were inverted overnight at 4°C. The bead supernatant was then removed. Beads were washed three times by suspending in 500 µL of washing buffer (10 mM Tris, 150 mM NaCl, 0.1 % (v/v) NP-40, 1 mM EDTA, pH 7.5), followed by pulling down with a magnet and supernatant aspiration. Finally, bound proteins were eluted from beads by adding 20 µL of a 25 mM solution of Biotin in washing buffer at 90 °C and boiling at 95 °C for 5 min. Eluted fractions and supernatants were analyzed by SDS-PAGE followed by Coomassie staining.

Titration *of N*-ethylmaleimide and lysis buffer additives for DUB inhibition in lysates

HEK293 cells were lysed in standard lysis buffer (50 mM Tris, 150 mM NaCl, 1% (v/v) NP-40, 1 mM EDTA, 5 % (v/v) glycerol, protease inhibitor cocktail, pH 7.5) and debris were removed by centrifugation for 10 min at 4 °C and 17 000 xg. The protein concentration was adjusted to 2 mg/mL. The lysate was split into the respective number of samples containing 100 µL each and *N*-ethylmaleimide (NEM) in concentrations from 0 to 20 mM or different additives for DUB inhibition were added. NEM was predissolved in 20x the desired concentration in EtOH and added immediately before lysis to the respective buffer solution. Afterwards, K63-linked triUb chains (8.4 mg/mL, 329 µM, 1 µL) were spiked into the lysates and the mixtures were incubated at 4 °C for 1 h. 4x LDS sample buffer (NuPAGE, Thermo Fischer Scientific, #NP0008) was added and samples were boiled at 95 °C for 5 min. Samples were analyzed by immunoblotting against ubiquitin as described below to identify the extend of cleaved Ub chains.

Mammalian cell culture

Jurkat cells were obtained from CLS Cell Line Service (#302147) and cultured in RPMI1640 medium (Pan Biotech, #P04-18047) supplied with 10 % fetal bovine serum (FBS, Gibco). Cells

were seeded in a density of 200 000 cells per mL and passaged every two to three days in a ratio of 1:10 (200 000 cells per mL). Cells were grown at 37 °C in 5 % CO_2 atmosphere with humidification. Cell counting was performed using a Countess II (Invitrogen) with trypan blue staining. Cells were established to be free from mycoplasma contamination every month using the MycoAlert® Mycoplasma Detection Assay (Lonza). HEK293 cells were cultured as described previously[2].

Small molecule treatment of Jurkat cells

7 x10^6 Jurkat cells were seeded into 2 mL of medium in 12-well plates. For proteasomal inhibition, cells were either pretreated with 0.5 µM of Carfilzomib (CFZ) for 1 h or as indicated. For TUBE pulldowns, Jurkat cells were treated with the indicated doses of small molecules for 4 h with or without proteasome or p97 inhibition as indicated. A 5 h treatment was used for NMS-873. For time course experiments, 750 000 Jurkat cells were seeded in 1 mL medium in a 24-well plate.

To investigate the time-dependent effect of proteasomal inhibition on BRD2 decay caused by MZ1, 10^6 Jurkat cells were seeded into 0.5 mL of medium into a 24 well plate. Afterwards, cells were treated with 500 nM of Carfilzomib at different timepoints, respectively DMSO at t=-60 min relative to the timepoint of the addition of 1 µM MZ1 to the cells, followed by a 4 h incubation time.

Cells were harvested by centrifugation at 500 xg for 5 min, washed with PBS and cell pellets were snap frozen in liquid nitrogen and stored at -80 °C until further use.

TUBE pulldown

Cells were lysed under denaturing conditions (4 M urea, 50 mM Tris, 150 mM NaCl, 1 % NP-40, 2 mM EDTA, 5 % glycerol, 20 mM NEM, protease inhibitor cocktail, pH 7.5) and incubated on ice for 30 min, followed by sonication in an ice-cooled sonicator bath (Bandelin Sonorex) for 4 x 30 s with 30 s breaks between intervals. Cell debris were removed by centrifugation at 17 000 to 20 000 xg for at least 10 min a 4 °C. Supernatants were transferred into fresh tubes and protein concentrations were determined with a DC assay using a BSA calibration curve. 30 µL of streptavidin magnetic beads (Pierce, #88817) per sample were transferred into a low-binding tube (Eppendorf, #022431081), were washed twice with washing buffer 1 (10 mM Tris, 150 mM NaCl, 0.1 % NP-40, 1 mM EDTA, pH 7.5) and resuspended in 100 µL of washing buffer 1 per sample. Afterwards, 10 µL of Biotin-TUBE (0.6 nmol) per sample were added and

the mixture was inverted for at least 1 h at 4°C. Beads were then washed to remove excess Biotin-TUBE and the equivalent of 30 µL of equilibrated beads were added into a fresh low-binding tube. Beads were pelleted and 1 mg of protein from lysates in 500 µL in lysis buffer was transferred to each tube. The mixture was inverted over night at 4 °C. Afterwards, beads were pelleted, and the supernatants were discarded. Beads were washed once using 1 mL of washing buffer 2 (4 M urea, 10 mM Tris, 150 mM NaCl, 0.1 % NP-40, 1 mM EDTA, pH 7.5), once with washing buffer 1 and two times with PBS, each time followed by gentle bead resuspension, pelleting of the beads and discarding of the supernatant.

For downstream immunoblot analysis, bound proteins were eluted from the beads using 20 µL of a 25 mM biotin solution in dilution buffer (saturated at 95°C) followed by heating and mixing of the samples for 5 min at 95°C. Beads were pelleted, the eluates were transferred into a fresh tube and elution was repeated as described before using 10 µL of 4x LDS (NuPAGE, Thermo Fischer Scientific, #NP0008). Finally, beads were pelleted, eluates were united (further concentrated through evaporation in the case of UBE3A ubiquitination analysis) and the eluted protein fractions were analyzed by western blot.

For proteomics analysis and to allow for separate elution of ubiquitinated proteins and the TUBE reagent, bound proteins were eluted twice by incubating the beads with 20 µL of a 100 mM glycine solution, pH 2.5, for 5 min at 37 °C, followed by bead separation. Both eluates were pooled and then neutralized with 3.6 µL of 1 M HEPES solution at pH 8.0. SP3 on-bead digest was performed as described previously, using 3 µL of SP3 bead mixture (50 µg/µL) per eluate resulting from 1 mg of cell lysate input for the TUBE pulldown[3]. For experiments shown in Fig. 2d, an additional boiling step with 4x LDS was performed as described above to elute TUBE reagent off the beads.

TUBE pulldown under different urea concentrations

Jurkat cells were lysed in 3 mL of 1.5x lysis buffer without urea for 1.5 hours at 4°C. Following this, the cells were sonicated in an ice-cooled water bath for 5x30 seconds, with 30-second breaks between intervals. Debris were removed by centrifugation at 17 000 xg for 40 min at 4°C. The protein concentration was determined by DC assay. 240 µL of streptavidin magnetic beads were equilibrated with 80 µL of Biotin-TUBE3 (4.8 nmol), as previously described, and subsequently washed and split into seven low-binding tubes. For each condition, 1 mg of cell lysate was diluted into 500 µL of 1x lysis buffer containing increasing concentrations of urea from 0 to 12 M (with precipitation observed in the 12 M sample). Lysate mixtures were added onto the TUBE-beads and inverted overnight at 4°C. The next day, the beads were pelleted and washed two times with washing buffer 1 and two times with PBS. The elution of bound

proteins from the beads was accomplished by subjecting them to boiling in 20 µL of a 25 mM biotin solution for five minutes, followed by bead separation and the transfer of the supernatant into a fresh tube. This procedure was repeated using 10 µL of 2x LDS sample buffer. The eluates were subsequently united, dried overnight in a speedvac, redissolved into 10 µL of MilliQ water, and analyzed by immunoblot against ubiquitin and β actin.

SDS-PAGE and Immunoblotting

For time course experiments, cells were lysed in standard lysis buffer (with 2 mM EDTA) and cleared lysates were prepared as described above. Samples were separated onto SDS Bis-Tris 4-12% gradient gels (NuPAGE, Thermo Fischer Scientific) and transferred onto 0.2 µm nitrocellulose membranes using either a semi-dry system (PierceG2 Fast Blotter, Thermo Scientific or Transblot Turbo, Bio-Rad) or an overnight wet tank transfer at 90 mA using towbin transfer buffer. Membranes were blocked using Intercept (PBS) Blocking Buffer (LI-COR Biosciences, #927-70001). Primary antibodies were diluted in Intercept T20 (PBS) Antibody Diluent (LI-COR Biosciences, #927-75001) and used as follows:

Antigen	Source organism	Supplier	Product number	Used dilution in WB
β-actin	Rabbit	Abcam (Cambridge, UK)	ab8227	1:2000
BRD2 (D89B4)	Rabbit	Cell Signaling Technology (Danvers, Massachusetts, USA)	5848S	1:1000
STK3	Rabbit	Proteintech Group, Inc (Rosemont, Illinois, USA)	12097-1-AP	1:500
UBE3A	Rabbit	Proteintech Group, Inc (Rosemont, Illinois, USA)	10344-1-AP	1:1000
Ubiquitin (FK2)	Mouse	Calbiochem Sigma-Aldrich (Burlington, Massachusetts, USA)	ST1200	1:1000
Ubiquitin (P4D1)	Mouse	Cell Signaling Technology (Danvers, Massachusetts, USA	3936S	1:1000
Vinculin	Mouse	Sigma-Aldrich (Burlington, Massachusetts, USA)	V9131	1:1000
Mouse IgG Secondary Antibody IRDye® 680RD	Goat	Li-Cor Biosciences (Lincoln, Nebraska, USA)	926-68070	1:1000
Mouse IgG Secondary Antibody IRDye® 800CW	Goat	Li-Cor Biosciences (Lincoln, Nebraska, USA)	926-32210	1:1000
Rabbit IgG, HRP-linked Antibody	Goat	Cell Signaling Technology (Danvers, Massachusetts, USA)	7074S	1:1000
Rabbit IgG Secondary Antibody IRDye® 800CW	Donkey	Li-Cor Biosciences (Lincoln, Nebraska, USA)	926-32213	1:1000

Membranes were incubated overnight at 4 °C with gentle rotation and washed three times for 5 min with 10 mL of PBS-T. Signal detection was performed by using secondary antibodies which were either coupled to HRP or fluorescently labeled (IRDye 680RD or IRDye 800RD, LI-COR Biosciences, see table above). If immunoblotting results were to be quantified, fluorescently labelled antibodies or HRP-coupled antibodies in combination with a substrate suitable for quantification (SuperSignal West Dura Extended Duration for quantification, Thermo Fisher Scientific, # 34075) were used for signal detection. Secondary antibodies were diluted in Intercept T20 (PBS) Antibody Diluent and incubated at RT for 1 h or at 4 °C for 3 h with gentle rotation. Finally, membranes were washed once briefly and three times for 10 min in PBS-T before being developed with Luminol substrate (SuperSignal West Femto #34095 or West Dura Extended Duration for quantification, Thermo Fisher Scientific, # 34075) or imaged by fluorescence using a ChemiDoc MP system (Bio-Rad). For quantitation, immunoblot images acquired on a ChemiDoc MP system were evaluated using the Biorad Image Lab software (V6.0.1). Adjusted volumes of the respective signal bands were exported and statistical inference including visualization of the data were performed in GraphPad Prism (V9.5.1).

Mass spectrometric analysis of pulldown eluates

SP3-digested samples were labeled using TMTpro 16plex Label Reagent Sets (Thermo Scientific, #A44522) or TMT10plex Isobaric Label Reagent Sets (Thermo Scientific, #90110) according to the manufacturer's protocol with half the amount of labeling reagent per sample and increased labeling times. In brief, 0.5 mg or 0.8 mg for 16plex or 10plex, respectively, of each isotope label were dissolved in 42 µL of dry ACN and 20 µL of label solution were added onto 100 µL of digested peptide solution. The labeling mixtures were incubated at RT for 2 h. Afterwards, the labelling reaction was quenched with hydroxylamine, 110 µL of each sample were pooled with the others, dried in a speed-vac and finally fractionated using a high pH Reversed-Phase Peptide Fractionation Kit (Pierce, #84868). Solvents were evaporated to dryness and sample were subjected to nanoHPLC-MS/MS analysis.

For nanoHPLC-MS/MS analysis samples were dissolved in 10 µL (10plex) or 20 µL (TMTpro 16plex) of 0.1% TFA in water and 10 µL (TMT10plex) or 3 µL (TMTpro 16plex) were injected onto an UltiMate 3000 RSLCnano system (Thermo Fisher Scientific, Germany) online coupled to a Q Exactive HF Hybrid Quadrupole-Orbitrap mass spectrometer equipped with a nanospray source (Nanospray Flex Ion Source, Thermo Scientific). All solvents were LC-MS grade. For desalting, the samples were injected onto a pre-column cartridge (5 µm, 100 Å, 300 µm ID x 5 mm, Dionex, Germany) using 0.1% TFA in water as eluent with a flow rate of

30 µL/min. Desalting was performed for 5 min with eluent flow to waste followed by back-flushing of the sample during the whole analysis from the pre-column to the PepMap100 RSLC C18 nanoHPLC column (2 µm, 100 Å, 75 µm ID x 50 cm, nanoViper, Dionex, Germany) using a linear gradient starting with 95% solvent A (water containing 0.1 % formic acid) / 5% solvent B (acetonitrile containing 0.1% formic acid) and increasing to 40% (TMT10plex) or 28% (TMTpro 16plex) solvent B within 120 min (TMT10plex) or 100 min (TMTpro 16plex), respectively, using a flow rate of 300 nL/min. Afterwards the column was washed (reaching 95% solvent B) and re-equilibrated to starting conditions. Spectra were recorded in data-dependent mode. For TMT10plex, a mass range of m/z 300 to 1650 was acquired with a resolution of 60000 for full scans, followed by up to 15 high energy collision dissociation (HCD) MS / MS scans of the most intense at least doubly charged ions using a resolution of 30000 and an NCE energy of 35%. For TMTpro 16plex, a mass range of m/z 375 to 1500 was acquired with a resolution of 120000 for full scan, followed by up to 15 high energy collision dissociation (HCD) MS / MS scans of the most intense at least doubly charged ions using a resolution of 60000 and an NCE energy of 32%. For samples corresponding to the data shown in Fig. 3c, the TMTpro 16plex-labelled samples were measured as described for TMT10plex-labelled samples with the injection volume being changed to 5 µL.

For samples corresponding to the data shown in Figure S5, LC-MS/MS analysis of peptide samples were performed on an Orbitrap Fusion Lumos mass spectrometer (Thermo Scientific, Waltham, MA, USA) coupled to a Vanquish Neo ultra high-performance liquid chromatography (UHPLC) system (Thermo Scientific, Waltham, MA, USA) that was operated in the one-column mode. The analytical column was a fused silica capillary (inner diameter 75 µm, outer diameter 360 µm, length 46 cm; CoAnn Technologies, Richland, WA, USA) with an integrated sintered frit packed in-house with Kinetex 1.7 µm XB-C18 core shell material (Phenomenex, Aschaffenburg, Germany). The analytical column was encased by a PRSO-V2 column oven (Sonation, Biberach, Germany) and attached to a nanospray flex ion source (Thermo Scientific, Waltham, MA, USA). The column oven temperature was set to 50 °C during sample loading and data acquisition. The LC was equipped with two mobile phases: solvent A (2% ACN and 0.2% FA, in water) and solvent B (80% ACN and 0.2% FA, in water). All solvents were of UHPLC grade (Honeywell, Charlotte, NC, USA). Peptides were directly loaded onto the analytical column with a maximum flow rate that would not exceed the set pressure limit of 950 bar (usually around 0.5 – 0.6 µL/min) and separated on the analytical column by running a 120 min gradient of solvent A and solvent B at a flow rate of 250 nL/min (start with 3% (v/v) B, gradient 3% to 9% (v/v) B for 5 min, gradient 9% to 28% (v/v) B for 87 min, gradient 28% to 100% (v/v) B for 16 min, 100% (v/v) B for 12 min).

The mass spectrometer was controlled by the Orbitrap Fusion Lumos Tune Application (v4.1.4244) and operated using the Xcalibur software (v4.7.69.37). For the experiment shown in Fig. S5, the mass spectrometer was set to: dynamic exclusion enabled (exclude after n times=1; exclusion duration (s)= 60; mass tolerance= ± 10ppm), intensity threshold: 5000, ion transfer tube temp: 250 °C and ion source voltage: 2500 V. In MS1, the analyzer was set to Fourier Transform (Orbitrap), max. Resolution at 200 m/z = 120000, scan range 400 – 1400 m/z, absolute automatic gain control = 400000, max. Ion acquisition time = 50 ms, RF Lens 30 % and data dependent mode (cycle time in seconds = 3 sec). In MS2, the analyzer was set to OT, max. resolution at 200 m/z = 120000, scan range: start 110 m/z, automatic gain control = 200%, absolute automatic gain control = 100000, max. Ion acquisition time = auto, charge states used for fragmentation: +2 to +7, Isolation mode: Quadrupole, Isolation window = 0.7 m/z, fragmentation method: higher-energy collisional dissociation and normalized collision energy = 35.

Analysis of proteomics data

Raw files from proteomics analysis were converted using the MSConvertGUI (64bit) package in ProteoWizard into mzML format, with applying the filter peakPicking vendor msLevel=1- [4]. Protein identification search and quantification of TMT-labeled samples were performed using FragPipe V20.0, V21.1 and V22.0 with MSFragger search engine, MSBooster and Percolator PSM rescoring, Philosopher FDR filtering and TMTintegrator quantification, using the default workflows for TMT10plex and TMTpro 16plex analysis provided by the program authors with specifying the used digestion enzymes as Trypsin and LysC[5-10]. For data sown in Fig. S5c, normalization was omitted (see caption) and the volcano plot was generated in Perseus (v2.0.7.0). All other proteomics data visualization was performed using FragPipe-Analyst (v0.35) and MSstasShiny[11]. For data shown in Fig. 4, annotation of known DUB and p97 targets was performed using BioGRID4.4. Therefore, hit proteins from proteomics experiments were searched within the respective subchapter of the deubiquitinase or p97. For data shown in Fig. S5d, protein assignment was performed using the Panther classification system. Additional sources for target annotation are cited in the respective sections of this publication.

Statistical analysis and reproducibility

For the quantification of protein abundance after western blot analysis (Fig. S6), the adjusted volume of bands corresponding to the protein of interest were quantified using ImageLab 6.0.1

(Bio-Rad). Vinculin-corrected intensity values of protein band adjusted volumes were normalized such that the mean DMSO intensity corresponds to 100%. Significance was assessed using unpaired t-tests.

For volcano plots, proteins in proteomics experiments were considered as significantly changed in abundance, if $-\log_{10}$ (p-value) was ≥ 2 (0.01) and $\log_2$ (fold change) ≤ –0.2 or ≥ 0.2 for cells without proteasomal inhibition or $\log_2$ (fold change) ≤ –0.5 or ≥ 0.5 for cells with proteasomal inhibition.

Treatments for proteomics analysis were performed in four biological replicates except for experiments shown in Fig. S5, which were performed in three biological replicates. Western blot-based experiments were conducted with typically three biological replicates unless stated otherwise. Optimization experiments shown in Fig. 2, Fig. S1 and Fig. S2 were conducted in one biological replicate, as the results were consistent with previous findings of highly similar experiments. The experiment shown in Fig. S6 was conducted with four biological replicates.

Data availability

Proteomics results are supplied as Table S1 (data shown in Fig. 3c), Table S2 (data shown in Fig. S4d), Table S3 (data shown in Fig. 4b-e) and Table S4 (data shown in Fig. S5c-e). Proteomics raw data are available via ProteomeXchange (ID: PXD057541 and PXD062845). Uncropped images of gels and blots are available in the Supporting Information.

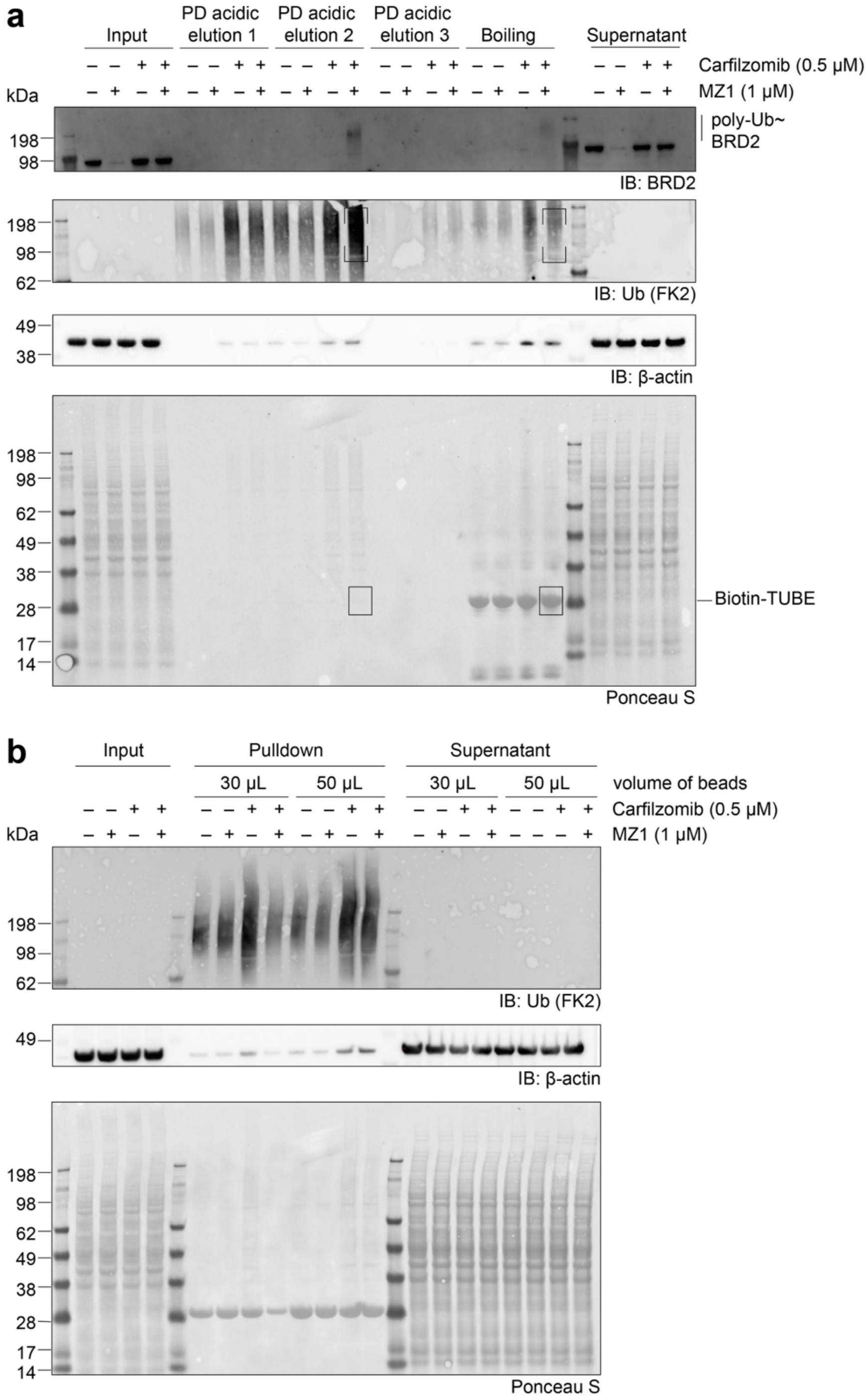

Figure S1. Pulldown optimization leads to enrichment of polyubiquitinated proteins with low background of unmodified proteins and separation of the TUBE reagent.

a. Western blots of TUBE pulldown (PD) fractions after three rounds of acidic elution (100 mM glycine, pH 2.5, elutions 1-3) and sequential boiling of the beads. Cropped images, indicated with boxes, are shown in Figure 2.

b. Assessment of different bead amounts to enrich ubiquitinated proteins from 1 mg of total protein in cell lysate. Beads were boiled to elute bound proteins for analysis.

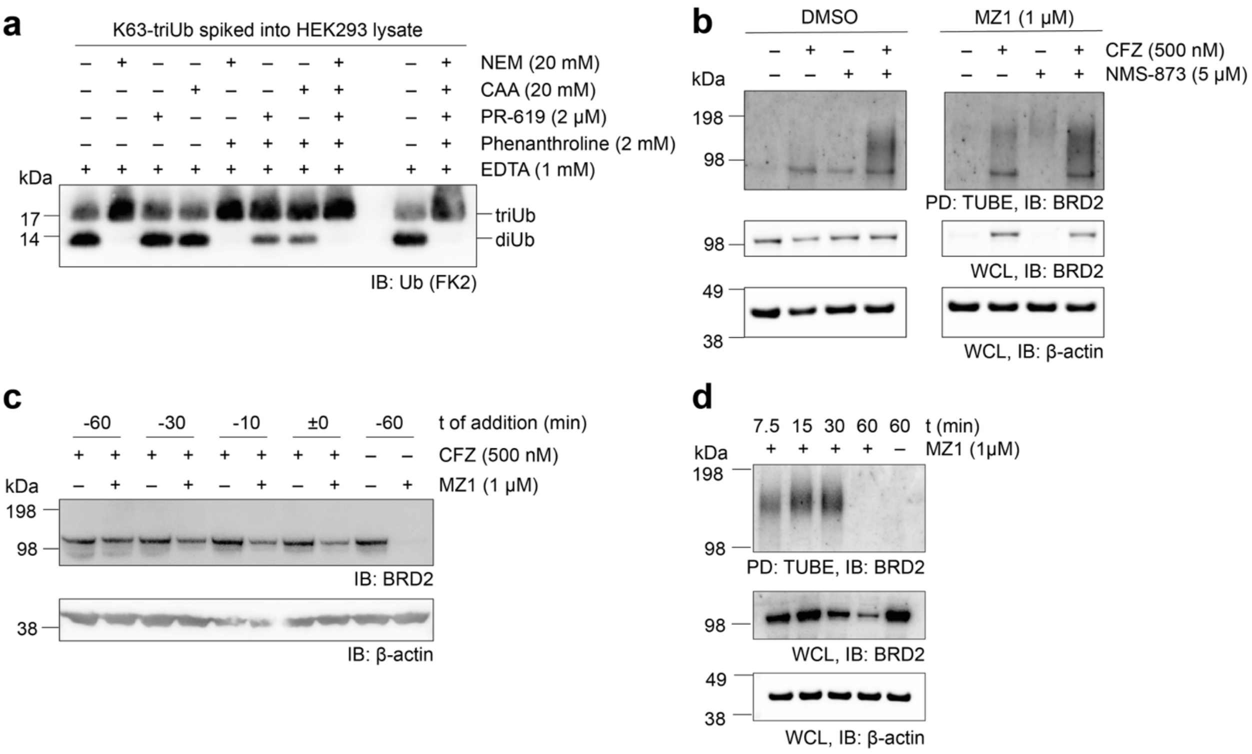

Figure S2. Optimization of cell treatment and lysis conditions.

a. Screening of different combinations of lysis buffer additives for preservation of Ub chains through DUB inhibition. K63-linked triUb chains were spiked into HEK293 lysate, samples were incubated for 1 h at 4°C and analyzed by immunoblotting.

b. TUBE pulldowns from Jurkat cells treated with proteasome inhibitor Carfilzomib (CFZ) and p97 inhibitor NMS-873 for 5 h, and MZ1 in the last 4 hours where indicated.

c. Timepoint titration of CFZ (0.5 µM) addition to investigate BRD2 levels. Jurkat cells were treated with MZ1 (1 µM) for 4 h, with CFZ having been added at indicated time points before.

d. TUBE pulldowns from Jurkat cells treated with MZ1 (1 µM) or DMSO as control for indicated durations for investigation of ubiquitination smear and degradation kinetics.

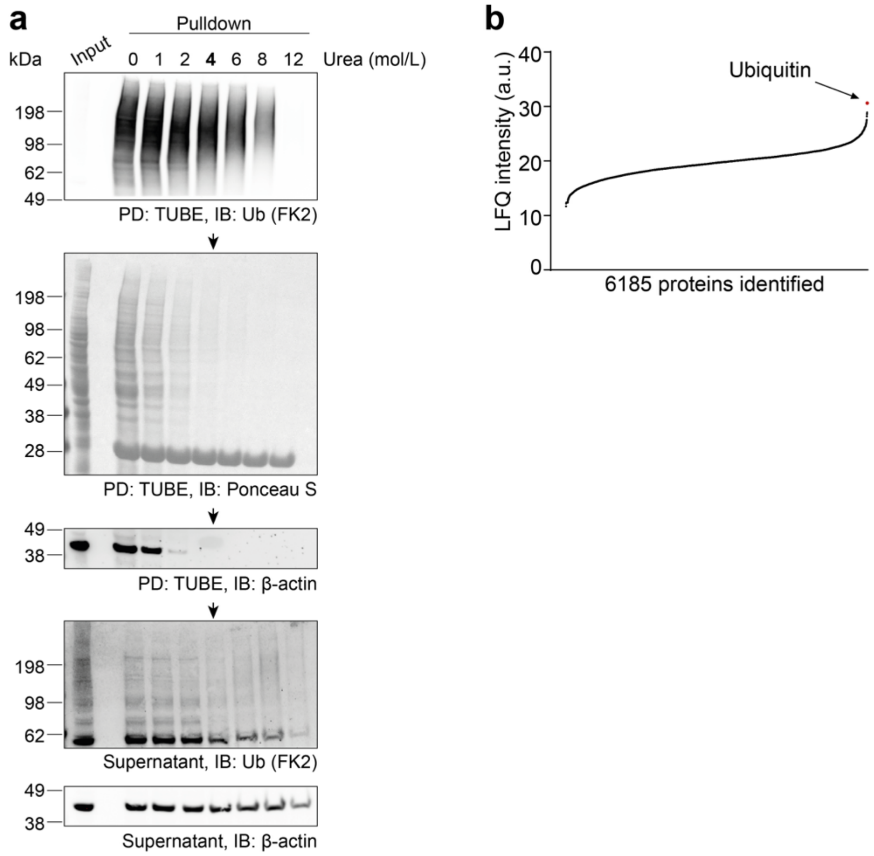

Figure S3. Titration of urea concentration during TUBE pulldown.

a. Screening of different concentrations of urea as indicated during the TUBE enrichment of polyubiquitinated proteins from Jurkat cell lysate. Arrows indicate the lanes of 4 M urea concentration during pulldowns as used as the standard lysis condition in this manuscript. The immunoblot against beta-actin of the TUBE-PD eluates was overexposed to visualize traces of beta-actin in the eluate fractions.

b. Depiction of LFQ intensities (mean of all 16 channels) of all proteins identified in the proteomics dataset shown in Fig. 3c, Fig. 4e and Fig. S4. Ubiquitin as the most abundant protein in the dataset is highlighted. Of the shown 6185 protein identified in this experiment, 6084 proteins were quantified without any missing values and were thus used for the subsequent analysis.

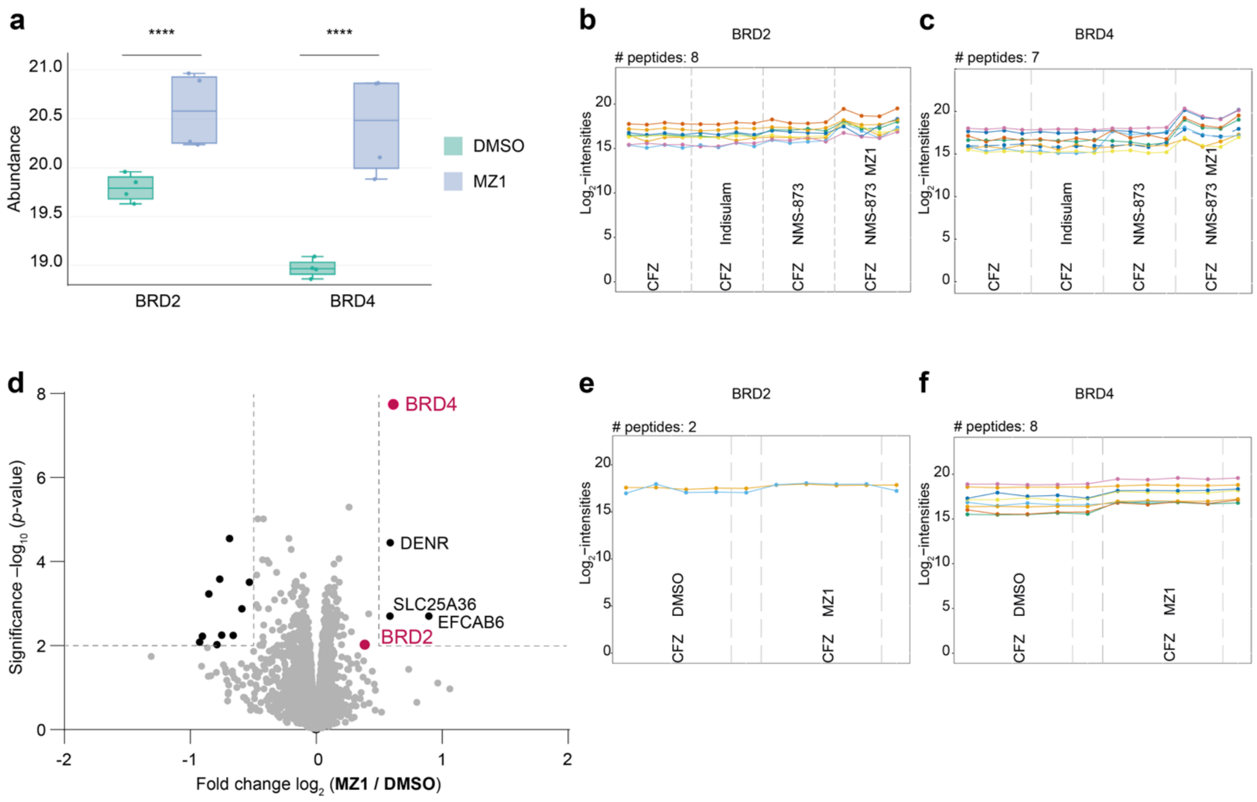

Figure S4. Mass spectrometric analysis of MZ1 degradation targets BRD2 and BRD4.

a. Profile plots of BRD2 and BRD4 abundance values with individual replicate values indicated (data from experiment shown in Fig. 3c, mean ± s.d.). Significance was assessed using unpaired t-tests. ****: $p < 0.0001$.

b.-c. Abundances of individual peptides in $\log_2$-scale for BRD2 (b) and BRD4 (c) for the experiment shown in Fig. 3c. Cells were treated with indicated compounds, and biological replicates were analyzed with TMTpro 16plex labeling.

d. Volcano plot of protein abundances after proteomic analysis of polyubiquitin pulldowns from Jurkat cells treated with MZ1 or DMSO. Experiment as in Fig. 3c, but with only CFZ and no NMS-873 treatment. MZ1 targets BRD2 and BRD4 are highlighted. Proteomics data were recorded in quadruplicate from independently treated cell samples and were analyzed using FragPipe as described in the methods section.

e.-f. Abundances of individual peptides in $\log_2$-scale for BRD2 (e) and BRD4 (f) in the dataset shown in panel d. Biological replicates were analyzed with TMT10plex labeling.

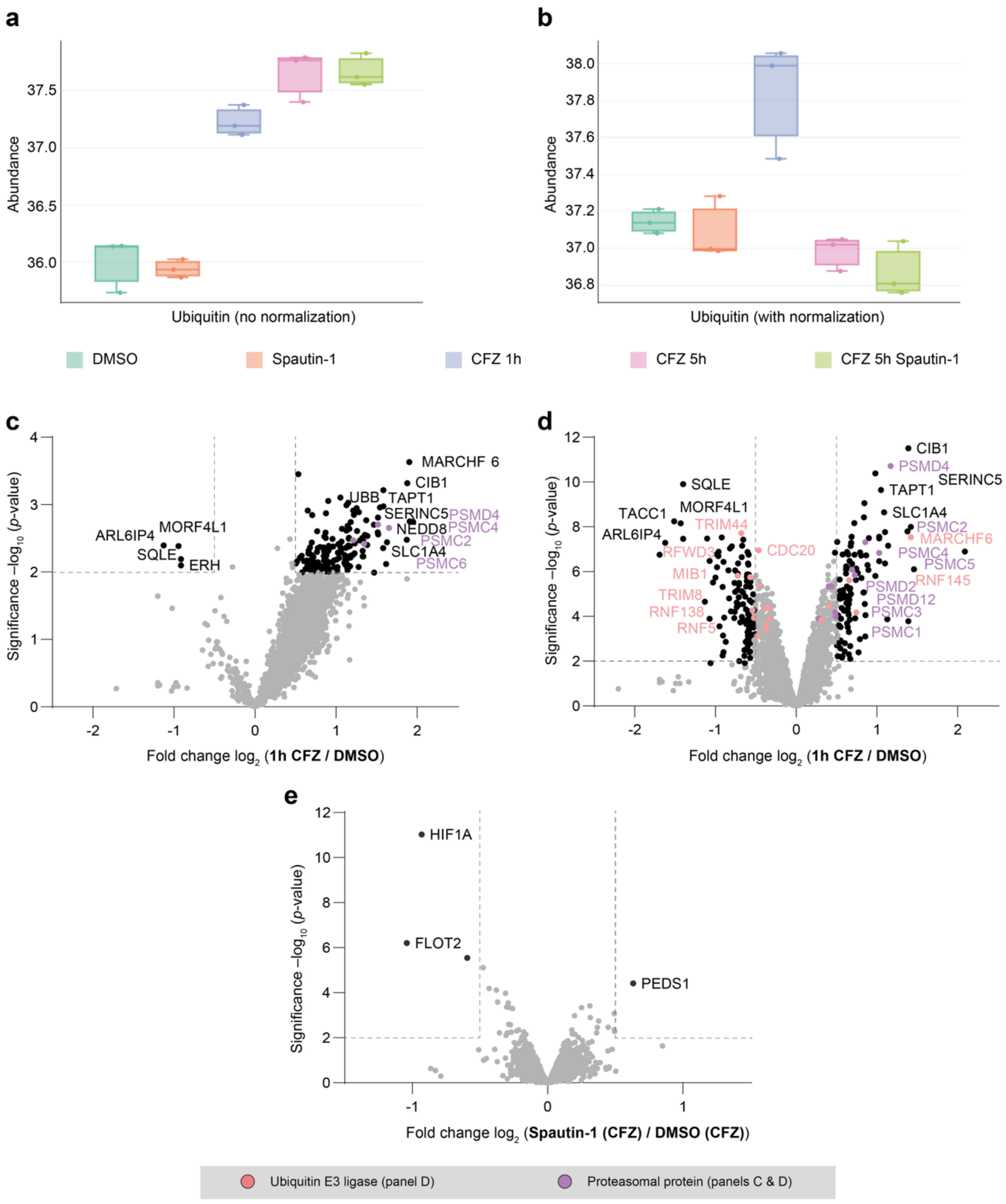

Figure S5. Mass spectrometric analysis of CFZ and Spautin-1-treated Jurkat cells.

a. Profile plots of Ubiquitin (UBB) abundance values without median-centered normalization with individual replicate values indicated (data from experiment shown in c, mean ± s.d.).

b. Profile plots of ubiquitin abundance values after median-centered normalization with individual replicate values indicated (data from experiment shown in d and e, mean ± s.d.)

c. Volcano plot of protein abundances after proteomic analysis without median-centered normalization of polyubiquitin pulldowns from Jurkat cells treated with Carfilzomib for 1 h

versus DMSO. Proteasomal proteins are highlighted. Among the less ubiquitinated proteins, MORFA4L1 was previously shown to be negatively impacted in its ubiquitination upon proteasome inhibition.[12]

d. Volcano plot of protein abundances after proteomic analysis with median-centered normalization of polyubiquitin pulldowns from Jurkat cells treated with Carfilzomib for 1 h versus DMSO. Ubiquitin E3 ligases and proteasomal proteins are highlighted.

e. Volcano plot of protein abundances after proteomic analysis with median-centered normalization of polyubiquitin pulldowns from Jurkat cells treated with Carfilzomib for 5 h and Spautin-1 for 4 h versus Carfilzomib for 5 h and DMSO for 4 h. Proteomics data were recorded in triplicates from independently treated cell samples and were analyzed using FragPipe as described in the methods section. Table S4 shows individual protein values depicted in this figure.

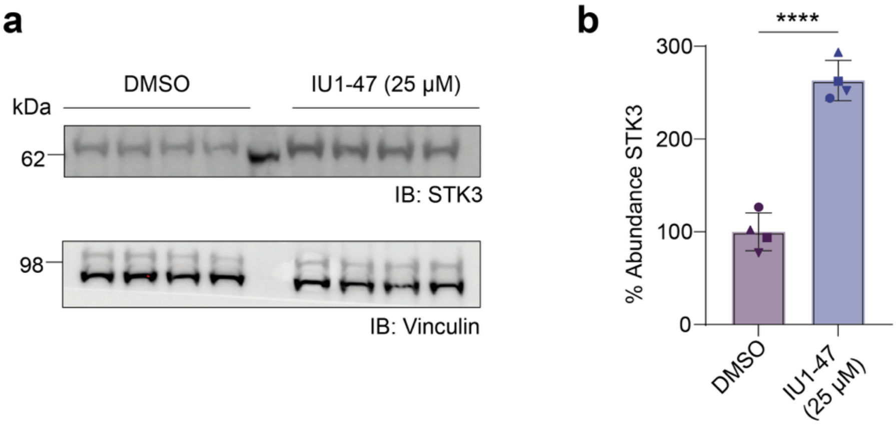

Figure S6. IU1-47 increases levels of STK3.

a. Immunoblotting of the Jurkat cell lysates used for PD proteomics (treated with USP14 inhibitor IU1-47 where indicated) for Hippo pathway protein STK3. Four replicates are shown per blot.

b. Densitometric quantification of results shown in panel a for STK3 (N = 4, mean ± s.d.). Vinculin-corrected intensity values were normalized to achieve mean abundances of DMSO-treated samples of 100%. Significance was assessed using unpaired t-tests. *P*-values are indicated with asterisks with a value below 0.05 considered a significant difference. ****: $p < 0.0001$.

Uncropped gels and blots (Page 1)

Figure 2c

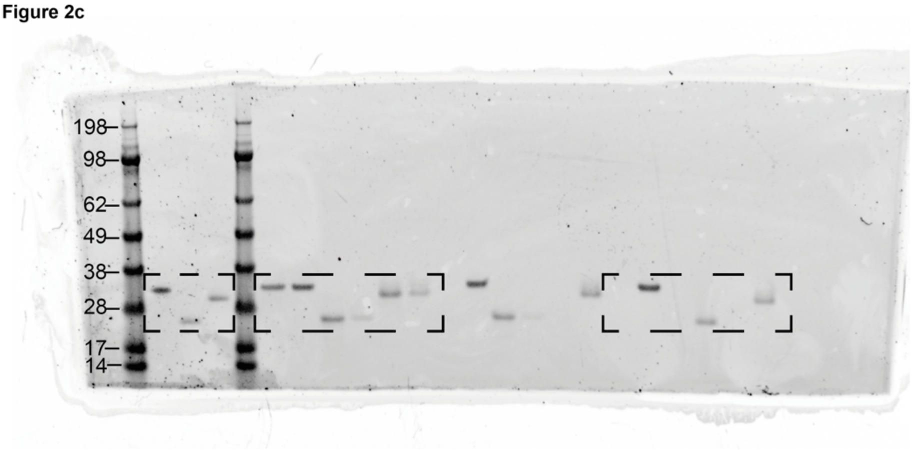

Figure 2e

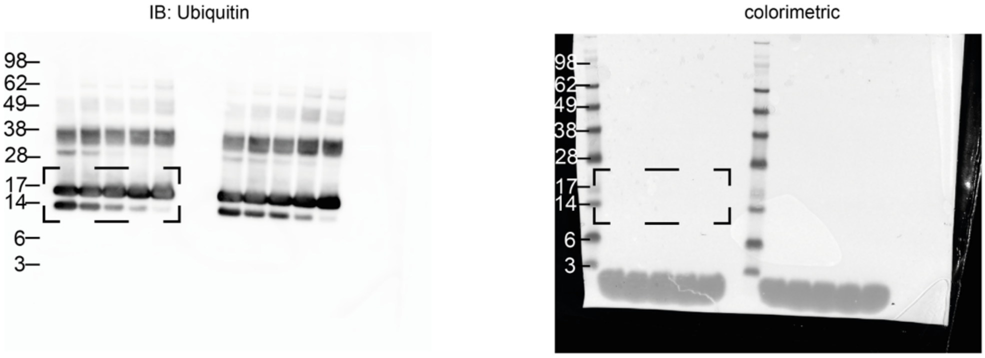

Uncropped gels and blots (Page 2)

Figure 3b

PD: TUBE, IB: BRD2

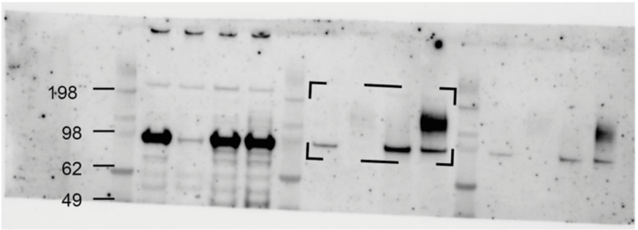

PD: Colorimetric

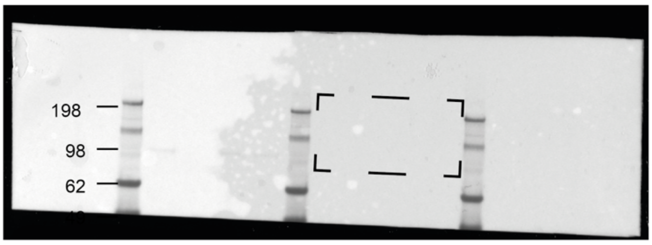

Input WCL, IB: BRD2

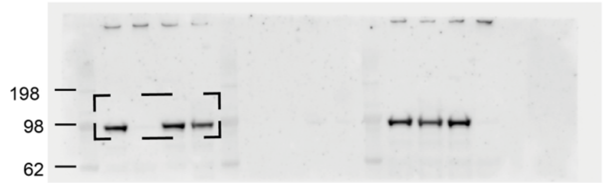

Input WCL: Colorimetric, inverted image display

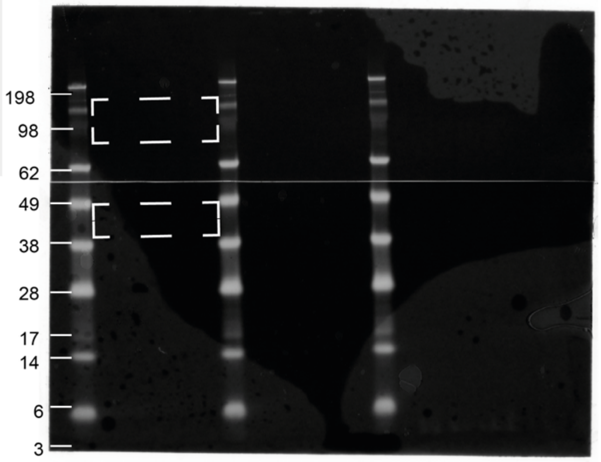

Input WCL, IB: β-actin

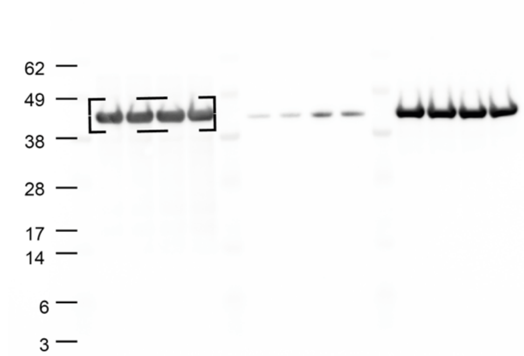

Uncropped gels and blots (Page 3)

Figure 5b

Input WCL, IB: UBE3A

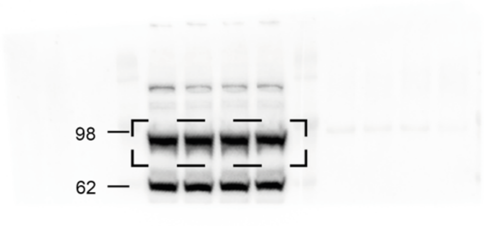

PD: TUBE, IB: UBE3A

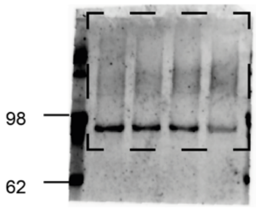

Input WCL, IB: β-actin

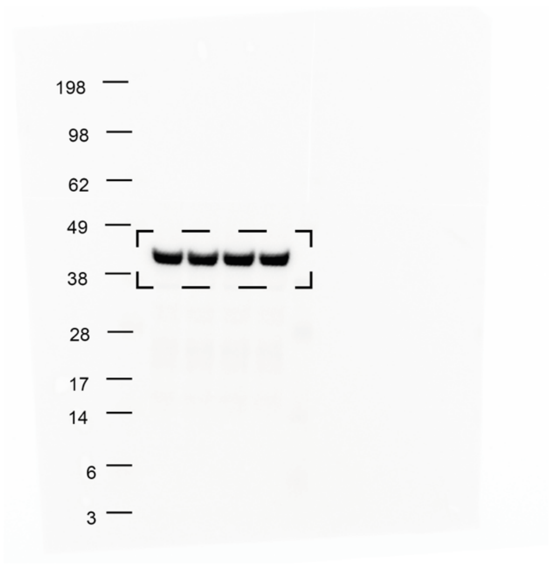

Colorimetric

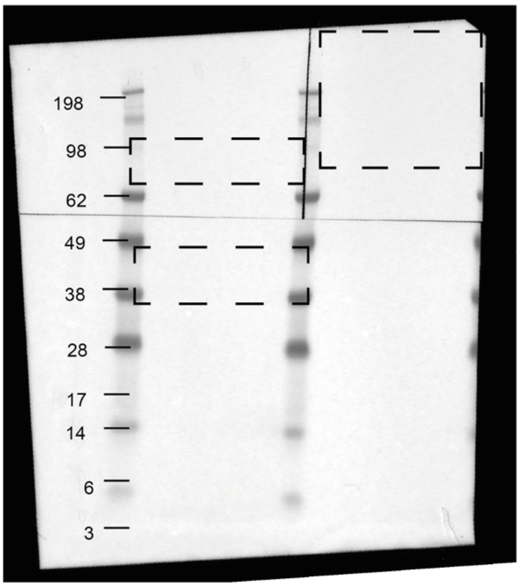

Uncropped gels and blots (Page 4)

Figure 5c

Colorimetric

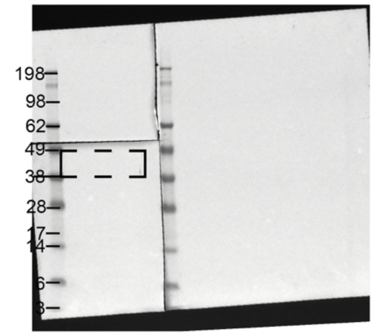

Input WCL, IB: β-actin

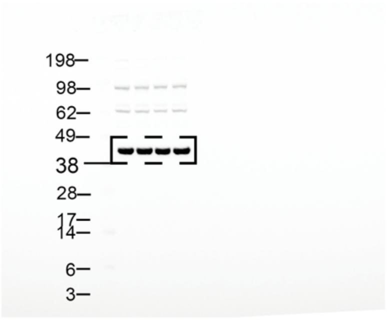

Colorimetric

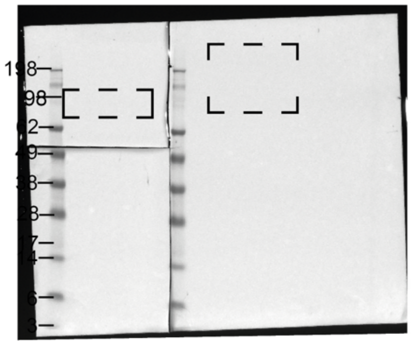

Input WCL, IB: UBE3A

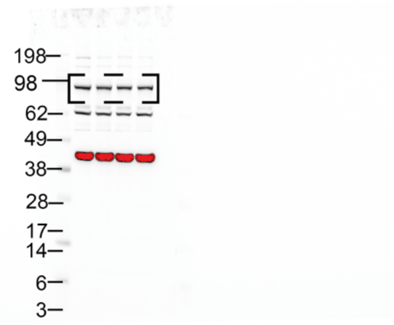

PD: TUBE, IB: UBE3A

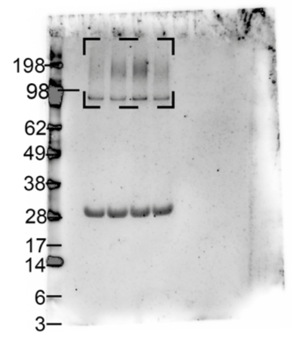

Uncropped gels and blots (Page 5)

Figure 5d

IB: UBE3A

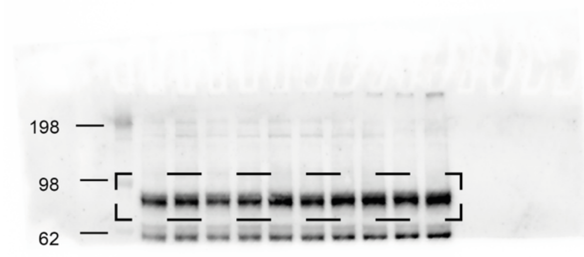

IB: β-actin

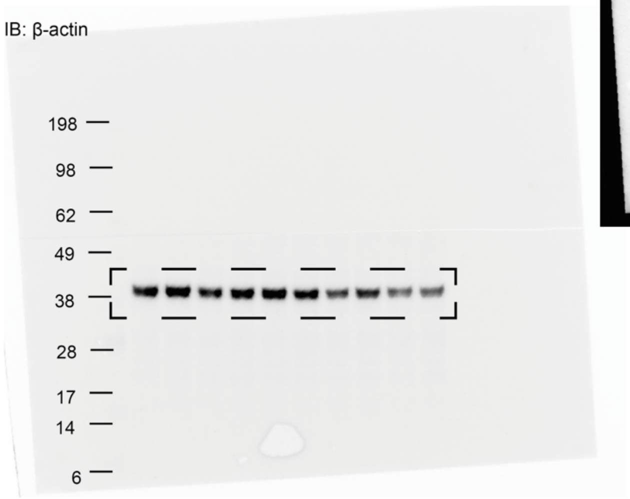

Colorimetric

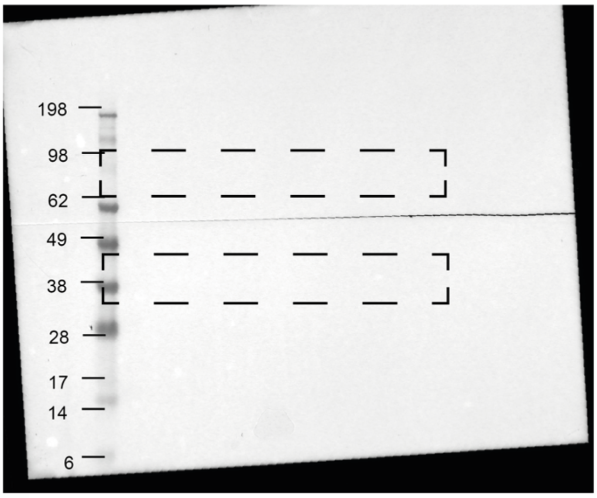

Uncropped gels and blots (Page 6)

Figure 2d + Figure S1a

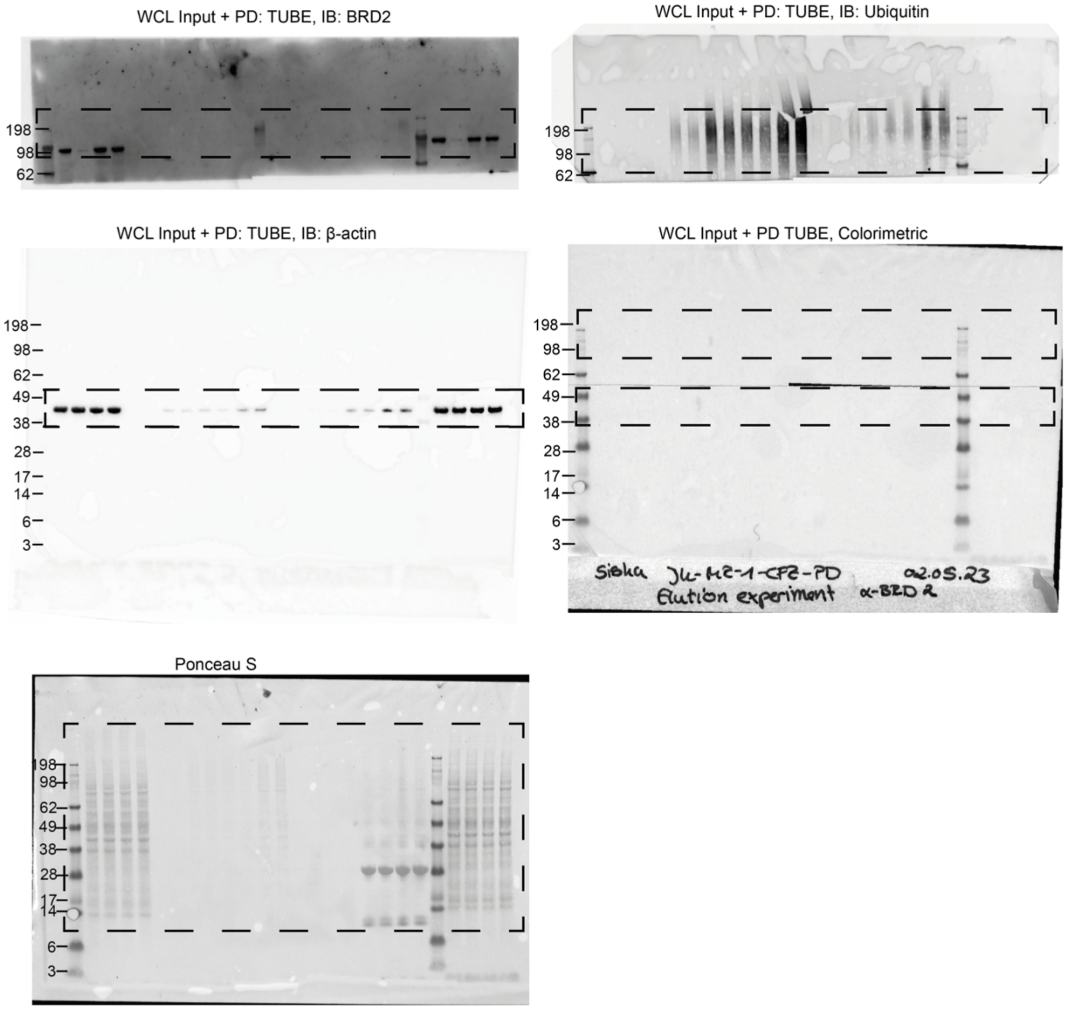

Uncropped gels and blots (Page 7)

Figure S1b

WCL Input + PD: TUBE, IB: Ubiquitin

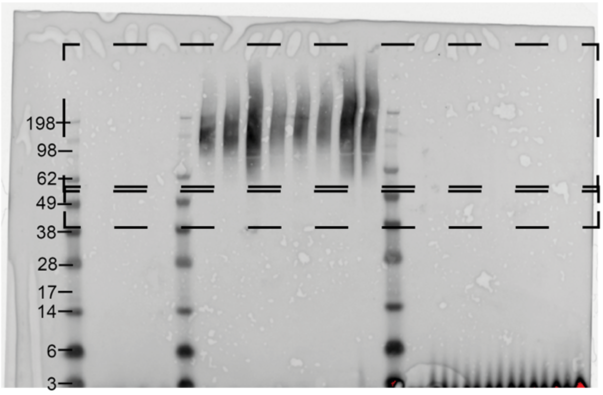

WCL Input + PD: TUBE, IB: β-actin

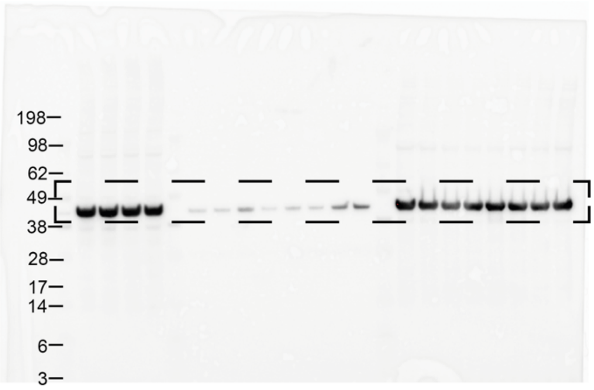

Ponceau S

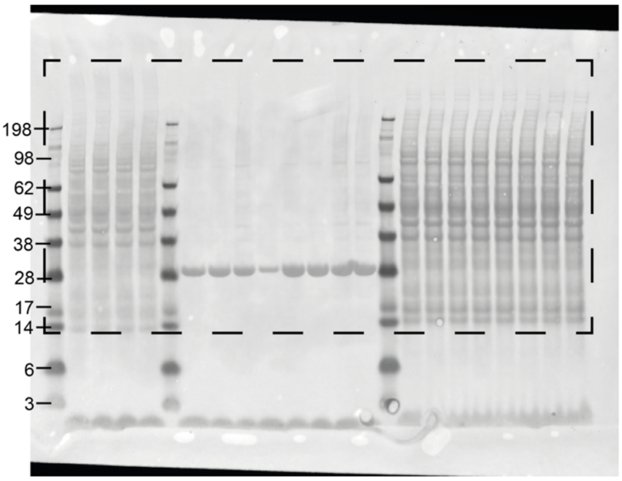

Uncropped gels and blots (Page 8)

Figure S2a

IB: Ubiquitin

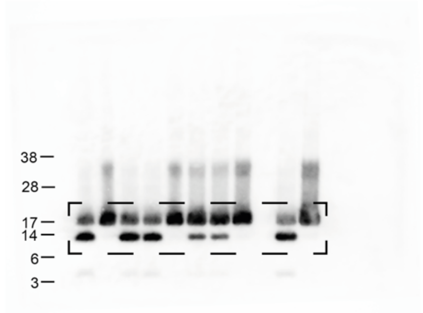

Colorimetric

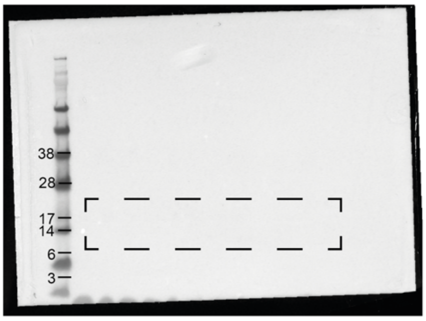

Figure S2c

IB: β-actin

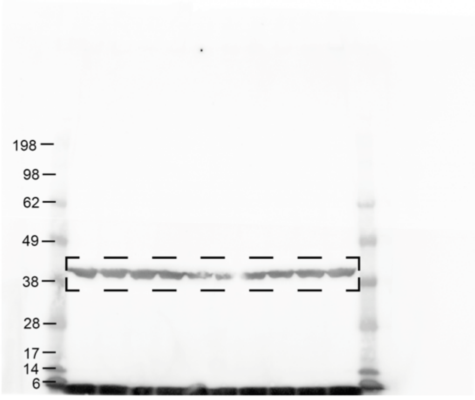

Colorimetric

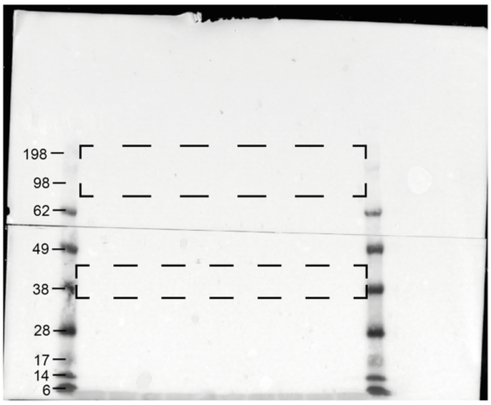

IB: BRD2

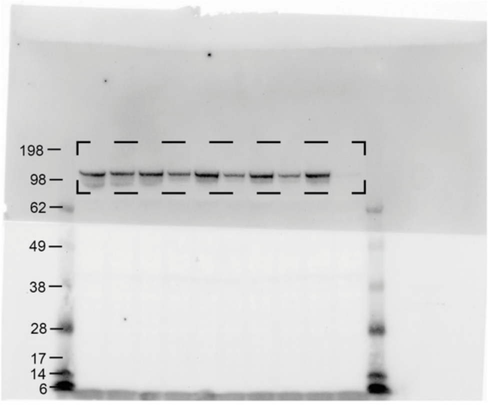

Uncropped gels and blots (Page 9)

Figure S2b

PD: TUBE, IB: BRD2

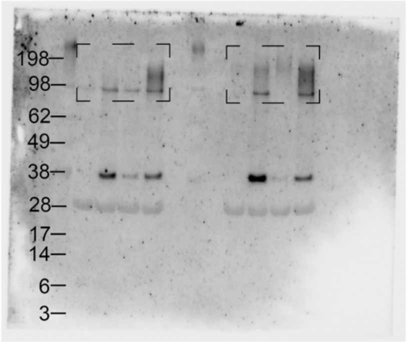

PD: Ponceau S

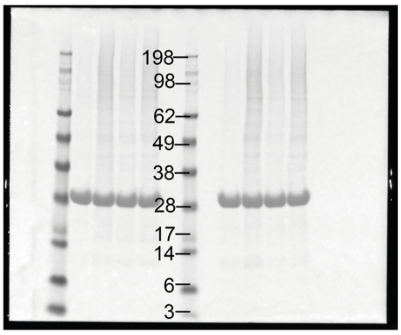

Input WCL, IB: BRD2

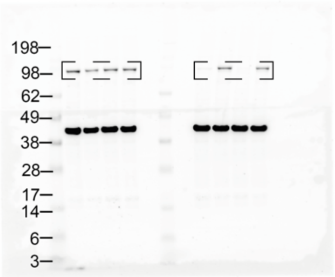

Input WCL: Colorimetric

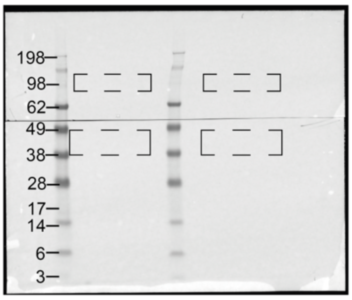

Input WCL, IB: β-actin

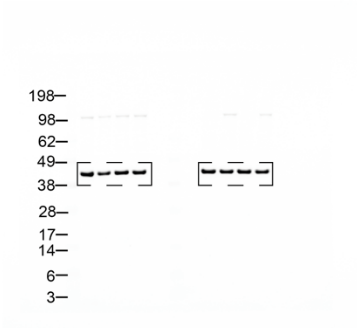

Uncropped gels and blots (Page 10)

Figure S2d

Colorimetric

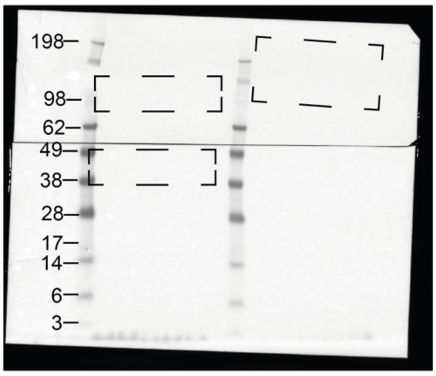

Input WCL and PD: TUBE, IB: BRD2

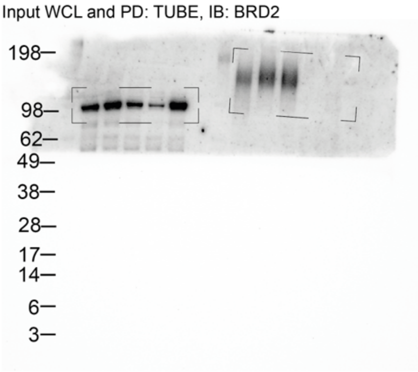

Input WCL, IB: β-actin

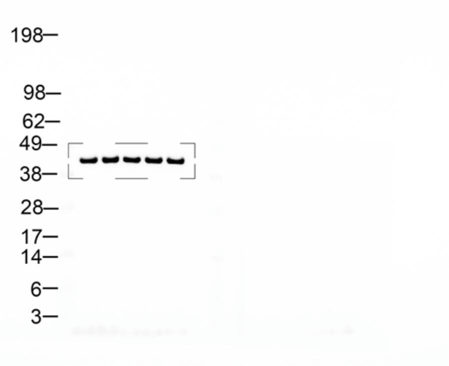

Uncropped gels and blots (Page 11)

Figure S3a

PD: TUBE, IB: Ponceau S

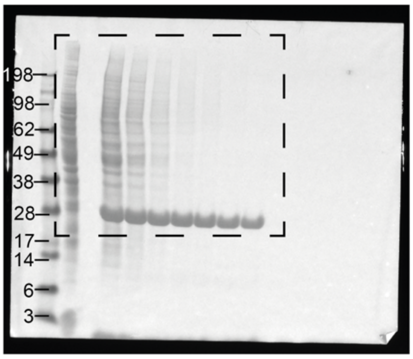

Supernatant, IB: Colorimetric

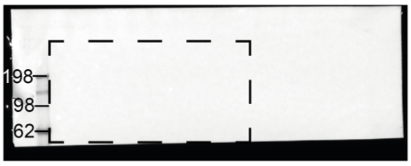

Supernatant, IB: Ub (FK2)

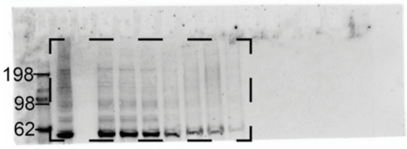

Supernatant, IB: Colorimetric

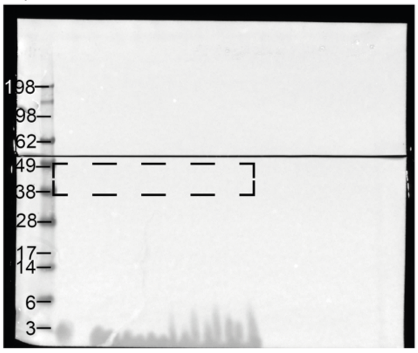

Supernatant, IB: β-actin

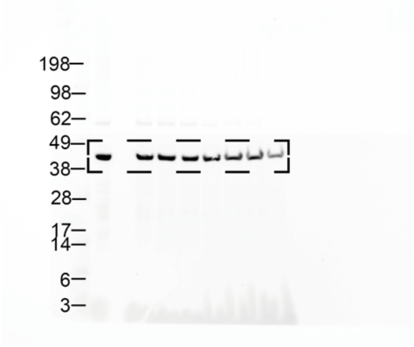

PD: TUBE, IB: Colorimetric

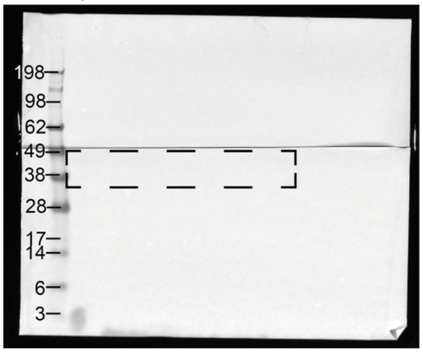

PD: TUBE, IB: β-actin (overexposed)

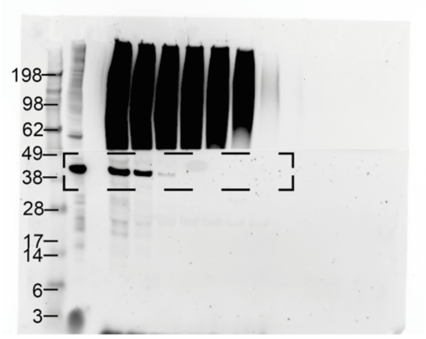

PD: TUBE, IB: Colorimetric

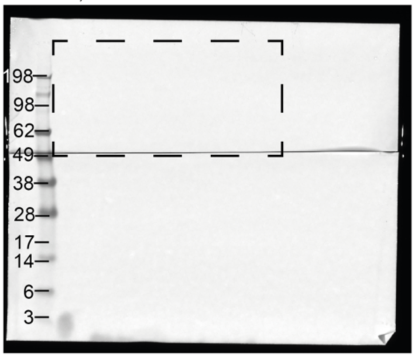

PD: TUBE, IB: Ub (FK2)

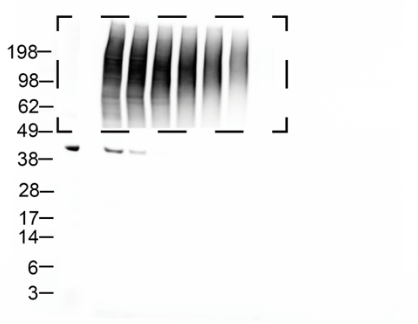

Uncropped gels and blots (Page 12)

Figure S6a

IB: STK3

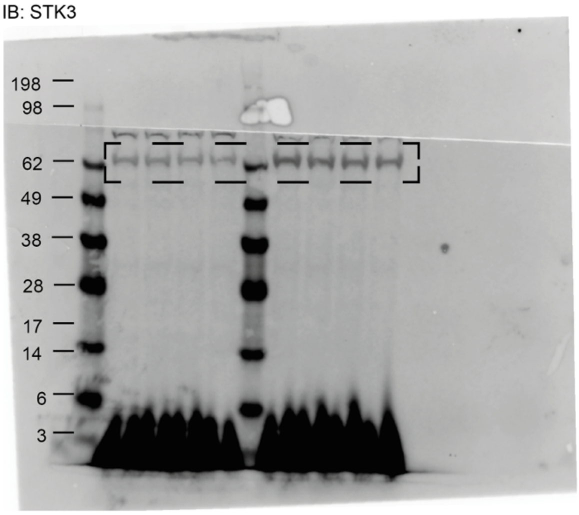

Colorimetric

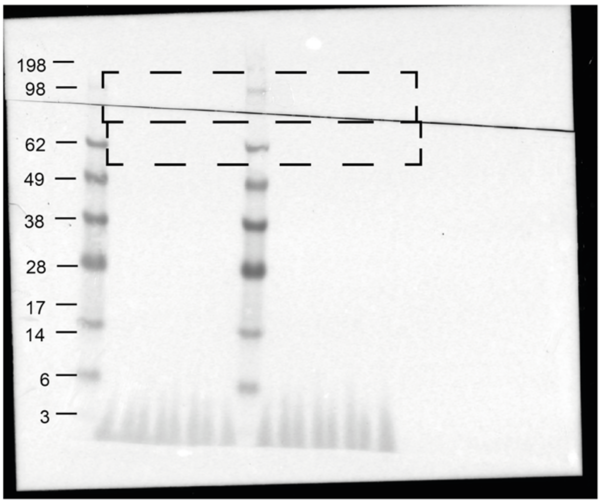

IB: Vinculin

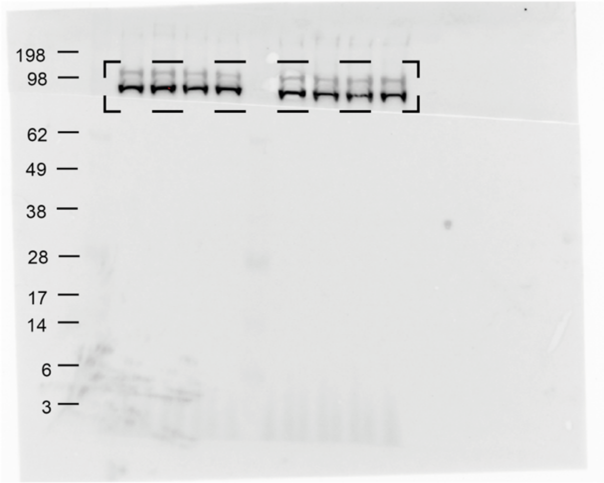

Supporting References

[1] R. Hjerpe, F. Aillet, F. Lopitz-Otsoa, V. Lang, P. England, M. S. Rodriguez, Efficient protection and isolation of ubiquitylated proteins using tandem ubiquitin-binding entities. *EMBO Rep* **2009**, *10*, 1250-1258.

[2] M. Schmidt, C. Grethe, S. Recknagel, G. M. Kipka, N. Klink, M. Gersch, N-Cyanopiperazines as Specific Covalent Inhibitors of the Deubiquitinating Enzyme UCHL1. *Angew Chem Int Ed Engl* **2024**, *63*, e202318849.

[3] C. S. Hughes, S. Moggridge, T. Muller, P. H. Sorensen, G. B. Morin, J. Krijgsveld, Single-pot, solid-phase-enhanced sample preparation for proteomics experiments. *Nat Protoc* **2019**, *14*, 68-85.

[4] J. D. Holman, D. L. Tabb, P. Mallick, Employing ProteoWizard to Convert Raw Mass Spectrometry Data. *Curr Protoc Bioinformatics* **2014**, *46*, 13 24 11-13 24 19.

[5] F. da Veiga Leprevost, S. E. Haynes, D. M. Avtonomov, H. Y. Chang, A. K. Shanmugam, D. Mellacheruvu, A. T. Kong, A. I. Nesvizhskii, Philosopher: a versatile toolkit for shotgun proteomics data analysis. *Nat Methods* **2020**, *17*, 869-870.

[6] L. Kall, J. D. Canterbury, J. Weston, W. S. Noble, M. J. MacCoss, Semi-supervised learning for peptide identification from shotgun proteomics datasets. *Nat Methods* **2007**, *4*, 923-925.

[7] A. T. Kong, F. V. Leprevost, D. M. Avtonomov, D. Mellacheruvu, A. I. Nesvizhskii, MSFragger: ultrafast and comprehensive peptide identification in mass spectrometry-based proteomics. *Nat Methods* **2017**, *14*, 513-520.

[8] A. I. Nesvizhskii, A. Keller, E. Kolker, R. Aebersold, A statistical model for identifying proteins by tandem mass spectrometry. *Anal Chem* **2003**, *75*, 4646-4658.

[9] G. C. Teo, D. A. Polasky, F. Yu, A. I. Nesvizhskii, Fast Deisotoping Algorithm and Its Implementation in the MSFragger Search Engine. *J Proteome Res* **2021**, *20*, 498-505.

[10] K. L. Yang, F. Yu, G. C. Teo, K. Li, V. Demichev, M. Ralser, A. I. Nesvizhskii, MSBooster: improving peptide identification rates using deep learning-based features. *Nat Commun* **2023**, *14*, 4539.

[11] D. Kohler, M. Kaza, C. Pasi, T. Huang, M. Staniak, D. Mohandas, E. Sabido, M. Choi, O. Vitek, MSstatsShiny: A GUI for Versatile, Scalable, and Reproducible Statistical Analyses of Quantitative Proteomic Experiments. *J Proteome Res* **2023**, *22*, 551-556.

[12] T. R. Porras-Yakushi, J. M. Reitsma, M. J. Sweredoski, R. J. Deshaies, S. Hess, In-depth proteomic analysis of proteasome inhibitors bortezomib, carfilzomib and MG132 reveals that mortality factor 4-like 1 (MORF4L1) protein ubiquitylation is negatively impacted. *J Proteomics* **2021**, *241*, 104197.
